# Dark-Blood Adiabatic *T*_*1ρ*_ Mapping of the Heart Using Combined Non-Selective and Slice-Selective RF Pulses at 3T

**DOI:** 10.3390/bioengineering13091031

**Published:** 2026-09-04

**Authors:** Chiara Coletti, Joao Tourais, Anastasia Fotaki, Yidong Zhao, Yi Zhang, Christal van de Steeg-Henzen, Qian Tao, Claudia Prieto, Sebastian Weingärtner

**Affiliations:** 1 Department of Imaging Physics, Delft University of Technology, 2628 CH Delft, The Netherlands; c.coletti@tudelft.nl (C.C.); j.l.silvacanaveiratourais@tudelft.nl (J.T.); y.zhao-8@tudelft.nl (Y.Z.); y.zhang-43@tudelft.nl (Y.Z.); q.tao@tudelft.nl (Q.T.); 2 Department of Biomedical Engineering, King’s College London, London WC2R 2LS, UK; anastasia.fotaki@kcl.ac.uk (A.F.); cdprieto@uc.cl (C.P.); 3 HollandPTC, 2629 JH Delft, The Netherlands; c.vande.steeg@hollandptc.nl; 4 School of Engineering, Pontificia Universidad Católica de Chile, Santiago 7820436, Chile; 5 Millenium Institute for Intelligent Healthcare Engineering, Santiago 7820436, Chile

**Keywords:** dark-blood, T_1*ρ* mapping_, spin-lock, adiabatic RF, myocardium

## Abstract

$T_{1\rho }$ mapping is emerging as a potential, contrast-free alternative to late gadolinium enhancement (LGE) for assessment of myocardial viability. However, strong signal contributions from the blood pool can impede quantitative evaluation at the (sub)endocardium. In this work, we study the effectiveness of dark-blood (DB) contrast in adiabatic $T_{1\rho }$ ($T_{1\rho ,adiab}$) mapping at 3T, using slice-selective and non-selective adiabatic spin-lock pulses. Adiabatic DB-$T_{1\rho ,adiab}$ preparations consisted of an odd number of slice-selective adiabatic full passage (AFP) pulses, followed by a final, non-selective AFP pulse. This preparation induces $T_{1\rho ,adiab}$ decay within the imaging slice while inverting the magnetization outside. A delay (δ) between preparation and imaging allowed for relaxation and inflow of the inverted blood to achieve DB contrast. Bias and precision of DB- and bright-blood (BB)-$T_{1\rho ,adiab}$ were compared in phantom and in healthy subjects (n = 10). Blood suppression efficacy and apparent myocardial thickness in DB imaging were investigated in simulations, phantom, and in vivo. The clinical feasibility of DB-$T_{1\rho ,adiab}$ mapping was evaluated in a small cohort of patients (n = 7) with suspected cardiovascular diseases. DB-$T_{1\rho ,adiab}$ values were in agreement with reference BB-$T_{1\rho ,adiab}$ values in phantom (myocardium-like vial BB: 219.27 ± 4.80 ms, DB: 218.09 ± 8.22 ms) and in healthy subjects (BB: 182.32 ± 28.27 ms, DB: 183.49 ± 45.54 ms). A moderate increase in intra-($wCV_{i,r}$) and inter-scan variability ($\bar{wCV_{i}}$) was observed in the DB method, compared with conventional BB imaging, for phantom and healthy subjects (in vivo $wCV_{i,r}$ BB: 15.51 ± 2.65%, DB: 24.82 ± 4.18%; in vivo $\bar{wCV_{i}}$ BB: 3.38 ± 0.86%, DB: 7.24 ± 2.55%). Longer delay times improved blood suppression in vivo for DB-$T_{1\rho ,adiab}$, albeit at increased intra-scan variability in phantom and in vivo (DB $wCV_{i,r}$ for δ = 0 ms: 4.90 ± 0.83% in phantom, 13.44 ± 2.91% in vivo, for δ = 600 ms: 8.14 ± 1.88% in phantom, 26.25 ± 5.19% in vivo). Average apparent myocardial thickness was slightly higher when using DB-$T_{1\rho ,adiab}$ compared with BB-$T_{1\rho ,adiab}$ (BB: 7.33 ± 2.05 mm, DB: 7.99 ± 2.46 mm). DB-$T_{1\rho ,adiab}$ maps yielded comparable image quality to BB-$T_{1\rho ,adiab}$ maps in patients. DB-$T_{1\rho ,adiab}$ mapping represents an alternative to BB-$T_{1\rho ,adiab}$ for myocardial assessment with the potential for improved visualization of the (sub-)endocardium.

## 1. Introduction

$T_{1\rho }$ mapping is gaining attention as a promising method for contrast-free myocardial tissue characterization and a potential alternative to late gadolinium enhancement (LGE) imaging [1]. While LGE remains the clinical gold standard for the assessment of fibrosis in ischemic and non-ischemic cardiomyopathies, the use of gadolinium-based contrast agents (GBCA) warrants caution. Gadolinium deposition, particularly in the brain, was observed in subjects following repeated contrast agent administration [2]. Moreover, GBCA can put patients with severe renal impairment at risk of nephrogenic systemic fibrosis [3]. Thus, non-contrast alternatives to LGE, such as those using quantitative parametric mapping techniques, are long sought after.

$T_{1\rho }$ mapping has shown promise as a non-contrast alternative due to its sensitivity to slow molecular motion and other interactions in the low kHz range [4,5]. $T_{1\rho }$ measures the longitudinal relaxation in the rotating frame of reference during irradiation with a radio-frequency (RF) pulse, the so-called spin-lock (SL) pulse. The SL field conventionally consists of moderate amplitude continuous-wave RF pulses, which lock the magnetization along the effective field and subject it to rotating-frame relaxation. Initial $T_{1\rho }$ mapping studies in animal models have reported improved contrast-to-noise ratio with respect to native $T_{1}$ and $T_{2}$ mapping for differentiating infarcted and remote myocardium [5,6,7,8,9,10]. In vivo $T_{1\rho }$ mapping has been successfully applied for scar assessment in patients with ischemic cardiomyopathies in several studies at 1.5T [11,12,13,14,15,16,17]. Applications of conventional $T_{1\rho }$ mapping at 3T are limited [18,19,20]. At this field strength $T_{1\rho }$ is highly susceptible to $B_{0}$ and $B_{1}^{+}$ inhomogeneities and requires high specific absorption rate (SAR) [21,22]. To overcome these limitations, adiabatic $T_{1\rho ,adiab}$ preparations [23,24] have been explored in cardiac imaging and have achieved robust $T_{1\rho ,adiab}$ mapping at 3T [25]. In $T_{1\rho ,adiab}$ the magnetization is locked by the effective field of a series of amplitude and frequency-modulated adiabatic full passage pulses (AFP), yielding a time-varying rotating-frame orientation and $T_{1\rho ,adiab}$ constant. Adiabatic pulses have been shown to be more robust than continuous-wave SLs in the presence of $B_{0}$ and $B_{1}^{+}$ inhomogeneities and require less SAR burden [25].

As commonly observed in myocardial tissue characterization, the ability to discern signal alterations at the sub-endocardium is compromised in $T_{1\rho }$ and $T_{1\rho ,adiab}$ maps due to the strong blood-pool signal and the limited resolution. In LGE imaging, this issue can be circumvented, using blood signal suppression in so-called, black-blood or dark-blood contrast [26,27]. Black-blood refers here to imaging techniques aimed at fully nulling the blood signal, while dark-blood techniques only attenuate the blood signal relative to the myocardium. In LGE imaging black- or dark-blood contrast has been previously achieved either through flow-dependent techniques, such as double inversion-recovery (DIR) preparations [28]. Alternatively, differences in relaxation times or magnetization transfer properties between blood, healthy myocardium and scar can be exploited for black-blood contrast [29,30,31,32,33,34,35,36,37,38,39]. Black-blood contrast has also been used in myocardial mapping. Black-blood $T_{1}$ mapping has previously been achieved with motion-sensitized driven-equilibrium (MSDE) preparations and demonstrated an increased thickness of the readily evaluable myocardium [40]. Black-blood $T_{2}$ and $T_{2}^{*}$ using DIR preparations have shown comparable or improved reproducibility and fewer artifacts, in addition to improved scar-to-blood contrast, compared with conventional bright-blood maps in vivo [41,42,43,44,45]. DIR preparations have also been used for black-blood imaging in continuous-wave $T_{1\rho }$ mapping, demonstrating improved visualization of sub-endocardial scar [46].

In this work, we seek to investigate dark-blood contrast in adiabatic $T_{1\rho ,adiab}$ mapping. A combination of selective and non-selective adiabatic pulses is proposed to achieve flow-dependent signal suppression of the blood pool with no additional preparations. Bloch simulations and phantom imaging were performed to characterize the bias of dark-blood $T_{1\rho ,adiab}$ preparations compared with reference bright-blood preparations. Furthermore, the influence of preparation parameters on blood suppression and intra-scan variability was studied. Finally, in vivo dark-blood $T_{1\rho ,adiab}$ mapping was tested in healthy subjects and in a small cohort of patients with suspected cardiovascular disease and compared with the corresponding bright-blood reference.

## 2. Materials and Methods

### 2.1. $T_{1\rho ,adiab}$ Preparations

**Figure 1:**
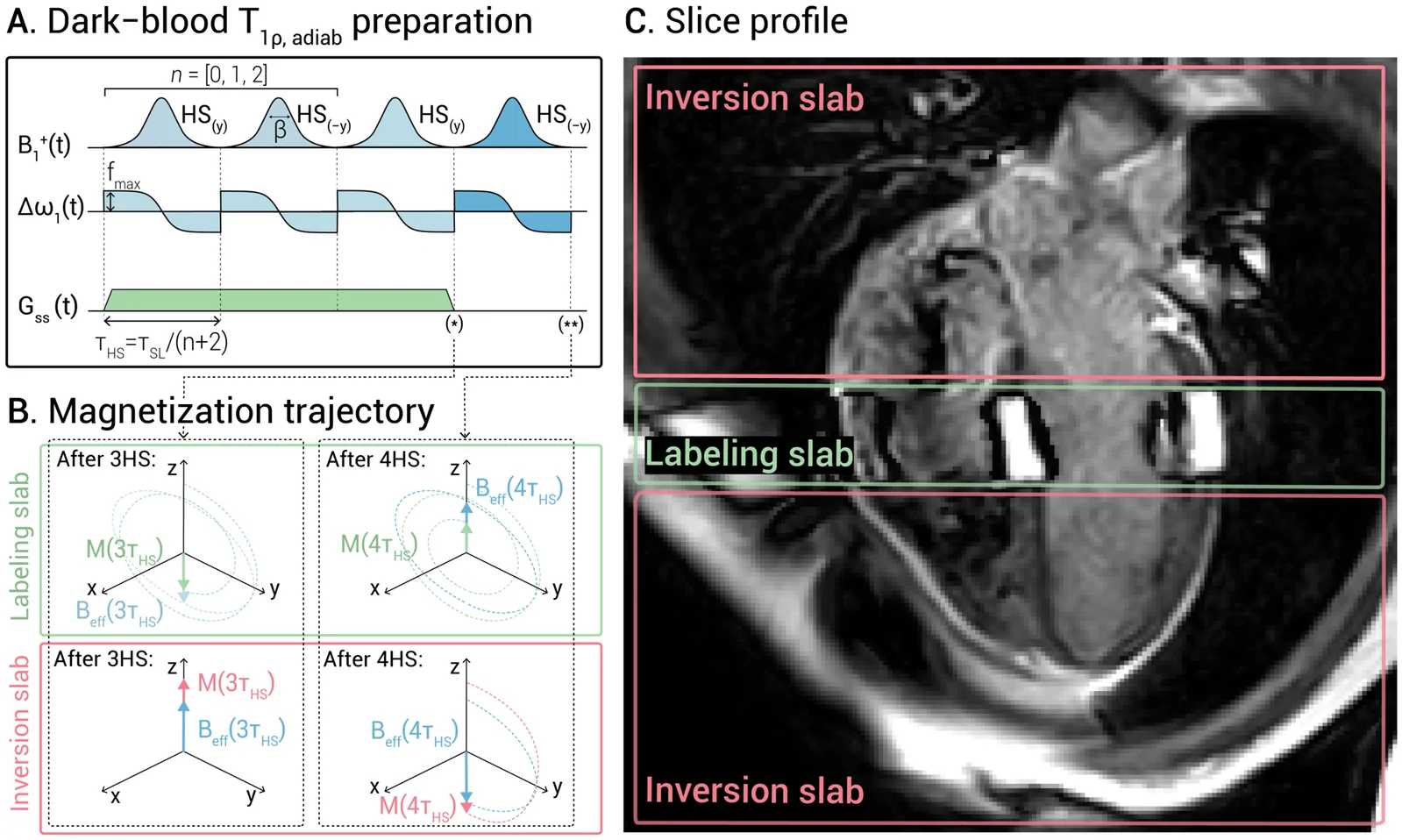
(**A**) $T_{1\rho ,adiab}$ preparation module, consisting of a train of hyperbolic secant (HS) pulses, with corresponding amplitude and frequency modulation functions. A slice-selective gradient ($G_{ss}$) is used on all but the last HS to achieve dark-blood (DB) contrast. (**B**) Magnetization (*M*) trajectories for spins in the labeling and inversion slabs during the slice-selective HS pulses (**left**) and at the end of the entire preparation module (**right**). Spins in the inversion slab only experience the last non-selective HS pulse and are effectively inverted at the end of the preparations, while spins in the labeling slab experience an even number of HS pulses, yielding a final $T_{1\rho ,adiab}$-prepared positive longitudinal magnetization. (**C**) Slice profile obtained with the DB-$T_{1\rho ,adiab}$-preparations, with $G_{ss}$ chosen orthogonal to the imaging slice orientation to visualize the labeling slab.

The proposed adiabatic spin-lock preparation comprises a train of hyperbolic secant (HS) AFPs (Figure 1A) [23,24,25]. In the preparation train, pairs of AFP were concatenated, ensuring that the magnetization is aligned with the +z direction at the end of the preparation. Each pair contained two HS pulses with alternating frequency sweep polarity to compensate for residual sensitivity to $B_{0}$ and $B_{1}^{+}$ inhomogeneities [25].

Dark-blood contrast was achieved by adding a slice-selection gradient for all HS pulses in the preparation train, except the last. This way, the labeling slab is exposed to an even number of AFPs, inducing $T_{1\rho ,adiab}$ decay (Figure 1B). Outside the labeling slab, only the last AFP is effective, leading to magnetization inversion (Figure 1B). A delay time δ between the preparation and the acquisition allows for the inverted blood to flow into the imaging slab. $T_{1}$ recovery of the inverted signal during the preparation delay enables the attenuation of the blood signal.

For bright-blood (BB) and dark-blood (DB) preparations, all HS AFP were played with a duration of $\tau_{HS}$ = 30 ms [25]. This value was chosen as a trade-off between adequate sampling of the expected range of in vivo $T_{1æ,\;\mathrm{adiab}}$ times and restrictions imposed by the SAR limits (whole-body SAR < 2.0 W/kg) and the RF amplifier chain. Width parameter β = 6.9 and frequency sweep amplitude $2\times f_{max}$ = 900 Hz were chosen, based on robustness optimization for the off-resonance range $\Delta \omega_{1}^{\mathrm{off}}\in \left\{-200,-199,\ldots +200\right\}$ Hz and relative $B_{1}^{+}$ values $\zeta_{1}\in \left\{0.75,0.76,\ldots 1.00\right\}$ [25]. In dark-blood preparations, the slab-selection gradient strength was set to 880 μT/m to achieve a labeling slab thickness of 24 mm, corresponding to three times the imaging slice thickness. The delay δ after the dark-blood preparation was adjusted according to the subject’s heart rate to allow for maximum $T_{1}$ recovery of the inverted signal, within a single heartbeat. No delay was played after the BB preparations.

### 2.2. $T_{1\rho ,adiab}$ Mapping Sequence

**Figure 2:**
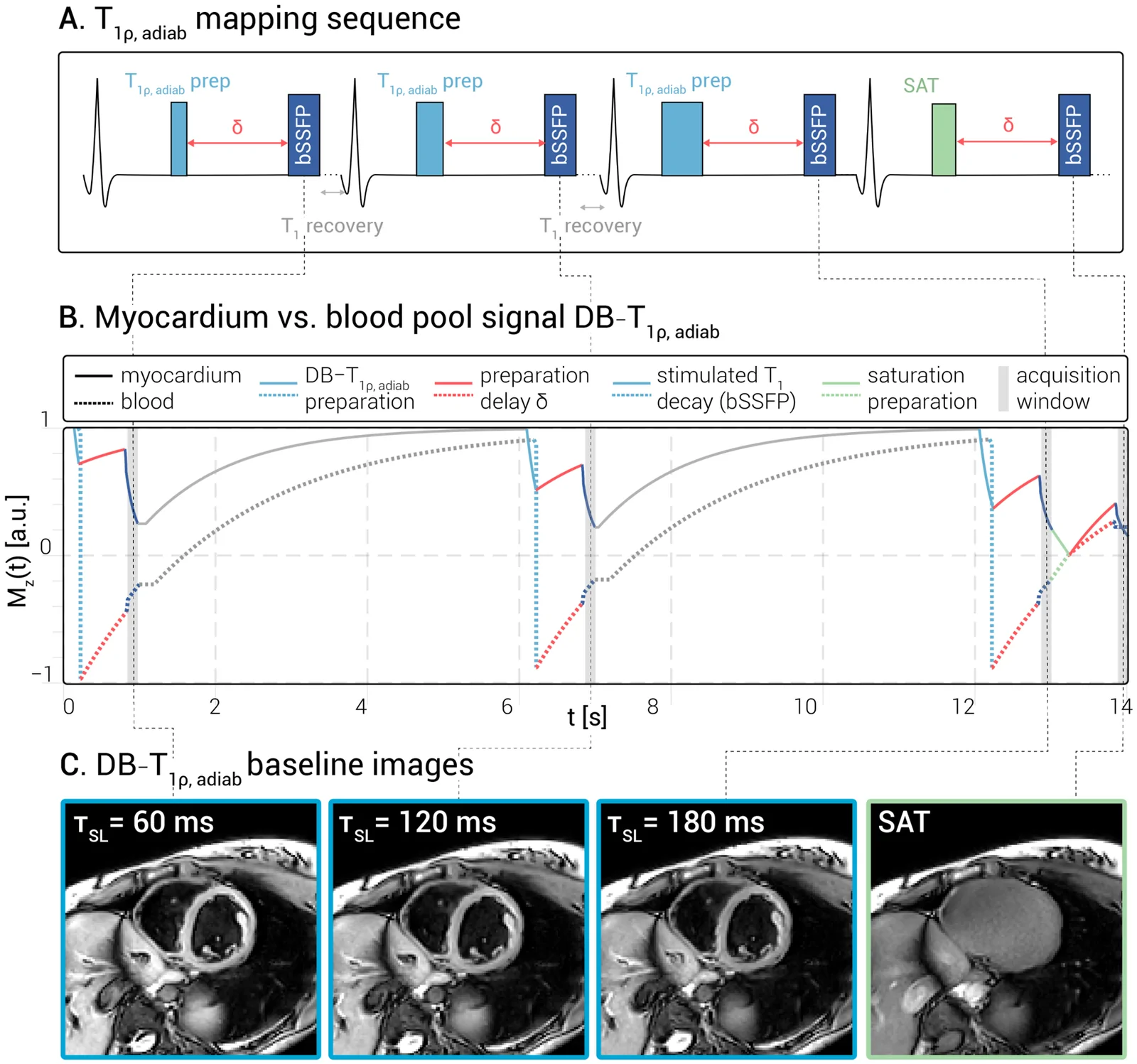
(**A**) Bright-blood (BB)- and dark-blood (DB)-$T_{1\rho }$ mapping sequence diagram. Four ECG-triggered end-diastolic bSSFP images were acquired in a single breath-hold. (**B**) Corresponding longitudinal magnetization profile ($M_{z}\left(t\right)$) for simulated myocardium (solid line, $T_{1}/T_{2}/T_{1æ,\mathrm{adiab}}$ = 1165/45/183 ms) in the labeling slab and simulated blood (dashed line, $T_{1}/T_{2}/T_{1æ,\mathrm{adiab}}$ = 1932/275/934 ms) in the inversion slab. (**C**) Corresponding DB-$T_{1æ,adiab}$ baseline images from a representative healthy subject. The first three images are acquired with increasing $T_{1æ,adiab}$ preparation duration ($\tau_{\mathrm{SL}}$ = 60, 120, 180 ms), while the fourth is preceded by a saturation preparation, to allow for unbiased DB-$T_{1æ,adiab}$ estimation.

Dark-blood preparations (DB-$T_{1\rho ,adiab}$) were compared to reference bright-blood preparations (BB-$T_{1\rho ,adiab}$) for adiabatic $T_{1\rho ,adiab}$ mapping using a 2D balanced steady-state free-precession (bSSFP) sequence (Figure 2). Four end-diastolic cardiac-triggered bSSFP baseline images were acquired in a single 14 s breath-hold. The first three baseline images were preceded by BB or DB-$T_{1\rho ,adiab}$ preparations of increasing length (2, 4, and 6 HS pulses, yielding $\tau_{SL}$ = 60, 120, 180 ms). The last baseline image was preceded by a composite “water suppression enhanced through $T_{1}$-effects” (WET) saturation pulse [47], to compensate for the effects of magnetization recovery during the imaging readout in the fitting procedure [48]. For DB-$T_{1\rho ,adiab}$ the saturation pulse was followed by the same preparation delay δ used in the $T_{1\rho ,adiab}$-prepared dynamics, to also capture the effect of $T_{1}$ recovery during the delay period. All images, except the saturation-prepared image, were preceded by a 5 s pause to allow for longitudinal magnetization recovery. The number of $T_{1\rho ,\;adiab}$-prepared baseline images was limited to three by the requirement to acquire all images within a single 14 s breath-hold at end-diastole. Other imaging parameters were in-plane resolution = 2 × 2 mm^2^, FOV = 220 × 220 mm^2^, slice thickness = 8 mm, TE/TR = 1.2/2.4 ms, flip angle = 70°, SENSE = 2, bandwidth = 1744 Hz/px.

BB and DB-$T_{1\rho ,adiab}$ maps were generated in MATLAB (R2022b) using the following three-parameter model [48], to account for the effect of the imaging pulses and magnetization recovery during the preparation delay:

(1) $$S\left(t\right)=A\cdot e^{-\frac{t}{T_{1æ,\mathrm{adiab}}}}+B.$$

Standard deviation maps were calculated alongside the parameter maps, based on the fit residuals, as described in Kellman et al. [49]. Thresholding was applied prior to the fitting procedure to focus the reconstruction on the region of interest. Voxels with a maximum intensity across all baseline images of less than 10% of the maximum signal intensity measured in the dynamic with the longest $T_{1\rho ,adiab}$ preparation were thresholded. Fits were considered failed if fitted $T_{1\rho ,\;adiab}$ values were non-finite, negative or more than 3 standard deviations away from the subject’s mean.

### 2.3. Experimental Evaluation

Phantoms and healthy subjects were scanned on a 3T Ingenia system (Philips, Best, The Netherlands), equipped with a 32-channel dS Anterior receive array, dual-channel parallel RF transmission with patient-adaptive B1 shimming, and second-order volume shimming. Patient data was acquired on a 3T Achieva system (Philips, Best, The Netherlands), equipped with a 32-channel cardiac phased-array receive coil, single-channel quadrature body-coil transmission, without full volume shimming. In vivo imaging was ethically approved by the competent review authorities (volunteers: METC NL73381.078.20, patients: UK National Research Ethics Service 15/NS/0030). Written informed consent has been obtained prior to all imaging sessions according to institutional guidelines.

#### 2.3.1. Phantom Validation

Phantom experiments were carried out with the T1MES phantom to mimic blood and myocardium relaxation times at 3T [50]. A preparation delay δ = 500 ms was used in phantom. Agreement between BB-$T_{1\rho ,\;adiab}$ and DB-$T_{1\rho ,\;adiab}$ was assessed with Bland–Altman analysis on the vial-wise mean $T_{1\rho ,\;adiab}$ values, reporting the bias (mean difference, DB-$T_{1\rho ,\;adiab}$ − BB-$T_{1\rho ,\;adiab}$) and the 95% limits of agreement (bias ± 1.96 SD of the differences). Ten repetitions of BB and DB-$T_{1\rho ,adiab}$ mapping scans were acquired to assess intra-scan and inter-scan variability. Manually drawn circular ROIs were used to extract $T_{1\rho ,adiab}$ values for subsequent statistical analysis.

Variability metrics were defined following the recommendations of the ISMRM Quantitative MR Study Group [51]. Within-acquisition precision was assessed for each vial *i* and repetition *r* as the ratio between the fit residuals’ standard deviation [49] and the average $T_{1\rho ,\;adiab}$ values:

(2) $$wCV_{i,r}=\frac{\sigma_{i,r}}{\mu_{i,r}}.$$

Here $\mu_{i,r}$ is the average $T_{1\rho ,adiab}$ within the ROI, while $\sigma_{i,r}$ is obtained by averaging the ROI values of the standard deviation maps. $wCV_{i,r}$ values were then averaged across repetitions to obtain the final intra-scan variability. Similarly, inter-scan variability across the *R* repetitions was quantified as the mean absolute relative deviation of the per-repetition ROI means ($\mu_{i,r}$) about their average ($\bar{\mu_{i}}$):

(3) $$\bar{wCV_{i}}=\frac{1}{R}\sum_{r=1}^{R}\frac{\left|(\mu_{i,r}-\bar{\mu_{i}})\right|}{\bar{\mu_{i}}},$$

where R=10 represents the number of repetitions. This definition adapts the conventional within-subject coefficient of variation by averaging per-repetition absolute deviations rather than pooling variances.

For statistical analysis, vials were grouped according to their $T_{1}$ regime into native myocardium-like (#4–6), post-contrast tissue-like (#1–3, #7 and #9) and blood-like (#8) groups [50]. Statistical differences between BB- and DB-$T_{1\rho ,\;adiab}$ were assessed using Wilcoxon signed-rank tests. A single test was performed for each group, with the paired vial- and repetition-wise coefficients as samples. Intra-scan variability was compared in all three groups. Inter-scan variability was only compared in the two multi-vial groups. As multiple tests were performed for each of these two metrics, both *p*-values were corrected using the Holm–Bonferroni adjustment. Both unadjusted and adjusted *p*-values are reported, and adjusted *p*-values of less than 0.05 were considered significant.

#### 2.3.2. In Vivo $T_{1\rho ,adiab}$ Mapping

Bright- and dark-blood $T_{1\rho ,adiab}$ maps were acquired in 10 healthy subjects (5 male, 5 female, 24.10 ± 6.08 y.o.) in 3 short-axis slices (apical, mid and basal). Three repetitions were acquired for each scan during a single session to study the reproducibility of BB- and DB-$T_{1\rho ,adiab}$ mapping. In a subset of subjects, four-chamber and two-chamber views were also acquired for both $T_{1\rho ,adiab}$ mapping techniques. The preparation delay δ was set to the maximum allowed value, ranging from 395 ms to 745 ms depending on each subject’s heart rate.

Image registration was carried out in a pairwise manner using normalized mutual information [52], applied identically to BB and DB data. The initial baseline image served as the reference template, and the remaining three baseline images were registered to this template. All registered image series were visually inspected for residual misalignment.

The myocardium was automatically segmented using the nnU-Net framework [53] with uncertainty estimation [54], applied as published without retraining or modification for this study. The network was trained on the publicly available ACDC dataset [55], containing annotated end-diastolic and end-systolic CINE volumes of 100 subjects. Segmentation maps with a predictive confidence below 75% were discarded and segmented manually; this was the case for 12 of 90 BB and 10 of 90 DB maps. The average BB- or DB-$T_{1\rho ,adiab}$ values and their standard deviation values (std) were extracted segment-by-segment in the myocardial ROI, following the 16-segments AHA model. Similarly to phantoms, agreement between myocardial BB-$T_{1\rho ,\;adiab}$ and DB-$T_{1\rho ,\;adiab}$ was assessed with Bland–Altman analysis on the subject-wise mean $T_{1\rho ,\;adiab}$ values. A paired Mann–Whitney U-test was performed on average BB and DB-$T_{1\rho ,adiab}$ values for each volunteer at a significance level of p<0.05.

BB- and DB-$T_{1\rho ,adiab}$ maps were compared in terms of intra- and inter-scan variability in a test–retest protocol. The within- ($wCV_{i,r}$) and across-repetitions coefficient of variability ($\bar{wCV_{i}}$) were computed segment-by-segment from the average $T_{1\rho ,adiab}$ and the standard deviation maps for each slice, subject, and repetition, as described for the phantom scans. $wCV_{i,r}$ coefficients were then averaged over all repetitions and subjects to calculate the final intra-scan variability coefficient for each myocardial segment. Similarly, each segment $\bar{wCV_{i}}$ value was averaged over all subjects to obtain the total inter-scan coefficient of variability. Additionally, the inter-subject variability was computed as the mean absolute relative deviation between each subject’s segment-wise average $T_{1\rho ,adiab}$ value and the overall segment average across all subjects:

(4) $$\bar{CV}=\frac{1}{N}\sum_{n\;=\;1}^{N}\frac{\left|(\mu_{i}-\bar{\mu })\right|}{\bar{\mu }}.$$

Here *N* indicates the number of subjects.

Statistical differences between BB- and DB-$T_{1\rho ,\;adiab}$ values, and between their intra-scan, inter-scan and inter-subject variability, were assessed using Wilcoxon signed-rank tests. Where possible, values were first averaged over repetitions and subjects, so that a single test was performed on the 16 paired segment-wise values, and no subject or repetition contributed more than one observation to any test. Four tests were performed in total, one for each quantity, and no correction for multiple comparisons was therefore required. The within- and between-subject variability averaged out in this step is quantified separately by the intra-scan, inter-scan and inter-subject variability metrics. The segment-wise comparisons are reported as descriptive analyses, characterizing the spatial consistency of the observed differences, rather than as independent confirmatory tests. *P*-values of less than 0.05 were considered significant.

#### 2.3.3. Blood Suppression

Additional experiments were performed to assess the effect of the preparation delay δ on blood suppression, accuracy, and variability of the $T_{1\rho ,adiab}$ quantification. Bloch simulations of the DB-$T_{1\rho ,adiab}$ mapping sequence were performed for myocardium-like tissue (simulated $T_{1}/T_{2}/T_{1æ,\mathrm{adiab}}$ = 1165/45/183 ms [56]) with varying preparation delay δ = 0, 10, …, 700 ms. Additive Gaussian noise was included to simulate SNR = 20. The same 3-parameter fit model described for phantom and in vivo mapping was used on simulated data. One hundred repetitions were computed for each preparation delay to estimate the DB-$T_{1\rho ,adiab}$ mean and standard deviation. The Bloch simulations modeled stationary spin compartments with complete inversion outside the labeling slab, and did not account for blood velocity, through-plane motion, incomplete inversion, or heart-rate variability. The simulations were designed to characterize the dependence of the $T_{1\rho ,\;adiab}$ estimate and its precision on the preparation delay, independently of flow-related behavior.

Phantom imaging was also performed for varying preparation delay δ = 0, 50, …, 700 ms. Mean and standard deviation were extracted from the DB-$T_{1\rho ,adiab}$ maps and the standard deviation maps of fit residuals for the manually segmented myocardium-like vial (#4: $T_{1}/T_{2}$ = 1260/48 ms [50]), respectively. DB-$T_{1\rho ,adiab}$ was then compared to the reference BB-$T_{1\rho ,adiab}$ mean value of the same vial (δ = 0 ms).

Finally, DB-$T_{1\rho ,adiab}$ maps for preparation delay δ = 0, 100, …, 600 ms were acquired in the mid-short-axis slice of a healthy volunteer (male, 27 y.o.). DB-$T_{1\rho ,adiab}$ mean and standard deviation were compared to reference values obtained for BB-$T_{1\rho ,adiab}$ mapping (δ = 0 ms) in the automatically segmented myocardial ROI.

The effect of increasing preparation delay on DB-$T_{1\rho ,adiab}$ mean and standard deviation was studied with linear regression analysis in simulations, phantom, and in vivo mapping. $R^{2}$ coefficient, slope, and intercept values were reported.

Blood suppression was additionally quantified on the baseline image acquired with the shortest $\tau_{SL}$ duration as the ratio between the mean signal intensity in a region of interest placed in the LV blood pool and the mean signal intensity in the myocardium. The metric was computed for BB and DB acquisitions in all healthy subjects for the mid-short-axis slice, and compared using a Wilcoxon signed-rank test.

#### 2.3.4. Myocardial Thickness

The apparent myocardial thickness in the presence of partial-voluming for BB- or DB-$T_{1\rho ,adiab}$ preparations was studied in simulations and in vivo. In Bloch simulations, partial-voluming was modeled at the signal level. For each blood volume fraction *f*, the baseline signals of the blood and myocardium compartments, $S_{b}\left(\tau_{SL}\right)$ and $S_{m}\left(\tau_{SL}\right)$, were obtained from independent Bloch simulations of the full preparation and readout, and combined as

(5) $$S\left(\tau_{SL}\right)=f\;S_{b}\left(\tau_{SL}\right)+\left(1-f\right)\;S_{m}\left(\tau_{SL}\right),$$

with f=0,0.05,…,1. Gaussian noise corresponding to an imaging SNR of 20 was added to the combined signal, which was then fitted with the same three-parameter model used for phantom and in vivo data. DB-$T_{1\rho ,adiab}$ was simulated for preparation delays δ = 0, 200, 400, 600 ms. Simulation parameters were $T_{1}/T_{2}/T_{1\rho ,\;adiab}=1165/45/183$ ms for myocardium and 1932/275/934 ms for blood. The resulting BB- and DB-$T_{1\rho ,adiab}$ values were averaged over one hundred simulation runs.

The performance of BB- and DB-$T_{1\rho ,adiab}$ mapping in voxels with blood/myocardium partial-voluming was also investigated in the healthy subject cohort. Automatically segmented myocardial ROIs in all three repetitions of the three short-axis slices were eroded or dilated from the inner border with a circular element of up to 6 mm radii to progressively exclude or include voxels with potential partial-voluming. BB- and DB-$T_{1\rho ,adiab}$ values for each erosion/dilation case were averaged first over the myocardial ROI and then across all slices, repetitions, and subjects. The final mean BB- and DB-$T_{1\rho ,adiab}$ values for each erosion/dilation case were reported with the standard deviation of their mean ROI values across slices, repetitions, and subjects.

Finally, the apparent myocardial thickness was compared between BB- and DB-$T_{1\rho ,adiab}$ short-axis maps for all healthy subjects. The thickness was estimated by considering 5° circular sectors centered in the central point of the segmented myocardium ROI. In these sectors, the number of voxels with $T_{1\rho ,adiab}$ values that lie within the ±15% interval around the mean myocardial $T_{1\rho ,adiab}$ value of each map was counted. The ±15% interval was chosen to match the deviation criterion used in the partial-volume analysis, where a blood fraction exceeding 5% caused a deviation of more than 15% in the BB-$T_{1\rho ,\;adiab}$ value. Voxels within this interval therefore correspond to myocardium that can be quantified without relevant blood contamination. The sector width of 5° was chosen as a trade-off between angular sampling and the requirement of at least one voxel per sector at the given in-plane resolution. The thickness was then averaged over all circular myocardial sectors to obtain the final myocardial thickness estimate. One-sided Wilcoxon signed-rank tests were used to assess whether DB-$T_{1\rho ,adiab}$ maps yielded larger apparent myocardial thickness than BB-$T_{1\rho ,adiab}$ maps. *p*-values smaller than 0.05 were considered significant.

#### 2.3.5. Patients $T_{1\rho ,adiab}$ Mapping

Clinical feasibility of BB and DB-$T_{1\rho ,adiab}$ mapping sequences was tested in a cohort of seven patients (2 male, 5 female, 52.80 ± 12.32 y.o.) referred to clinical CMR. Patients were imaged using clinical protocol sequences, including native MOLLI $T_{1}$ mapping (in-plane resolution = 1.1 × 1.1 mm^2^, FOV = 350 × 350 mm^2^, slice thickness = 10 mm, TE/TR = 0.99/2.14 ms, SENSE = 2), native Gradient Spin Echo (GraSE) $T_{2}$ mapping (in-plane resolution = 1.4 × 1.4 mm^2^, FOV = 220 × 220 mm^2^, slice thickness = 8 mm, TE/TR = 92/967 ms, SENSE = 2), and LGE imaging (inversion recovery spoiled Gradient Echo, PSIR, in-plane resolution = 0.7 ×0.7 mm^2^, FOV = 320×320 mm^2^, slice thickness = 8 mm, TE/TR = 2.0/3.50 ms, SENSE = 2), performed 10–15 min after injection of 0.15 mmol/kg of Gadobutrol (Gadovist, Bayer Schering, Berlin, Germany). BB- and DB-$T_{1\rho ,adiab}$ mapping were performed before contrast administration in a single mid-short-axis slice in all patients and a four-chamber view in four of the seven patients. The same image registration used for healthy subjects was applied to the $T_{1\rho ,adiab}$ maps of patients. Manual segmentation was performed to extract myocardial $T_{1\rho ,adiab}$, $T_{1}$ and $T_{2}$ in the septum. After review of all parametric maps acquired for each subject, examinations were excluded from the analysis if excessive motion artifacts or poor shimming corrupted all acquired quantitative maps.

## 3. Results

### 3.1. Phantom Validation Results

In visual assessment, phantom $T_{1\rho ,adiab}$ maps obtained with the BB and DB techniques appear free from artifacts and with homogeneous quantification within the vials. Quantitative analysis showed good agreement between DB-$T_{1\rho ,adiab}$ and BB-$T_{1\rho ,adiab}$ values (BB-$T_{1\rho ,adiab}$ = 219.27 ± 4.80 ms, DB-$T_{1\rho ,adiab}$ = 218.09 ± 8.22 ms for the myocardium-like vial #4, BB-$T_{1\rho ,adiab}$ = 893.94 ± 51.81 ms, DB-$T_{1\rho ,adiab}$ = 902.49 ± 55.93 ms in the blood-like vial #8). Across all nine vials, Bland–Altman analysis of the per-vial averages yielded a bias of +2.57 ms (95% CI −5.35 to +10.48 ms) with 95% limits of agreement of −17.61 to +22.74 ms, corresponding to −5.2% to +5.1% of the measured values (Figure S2A).

**Figure 3:**
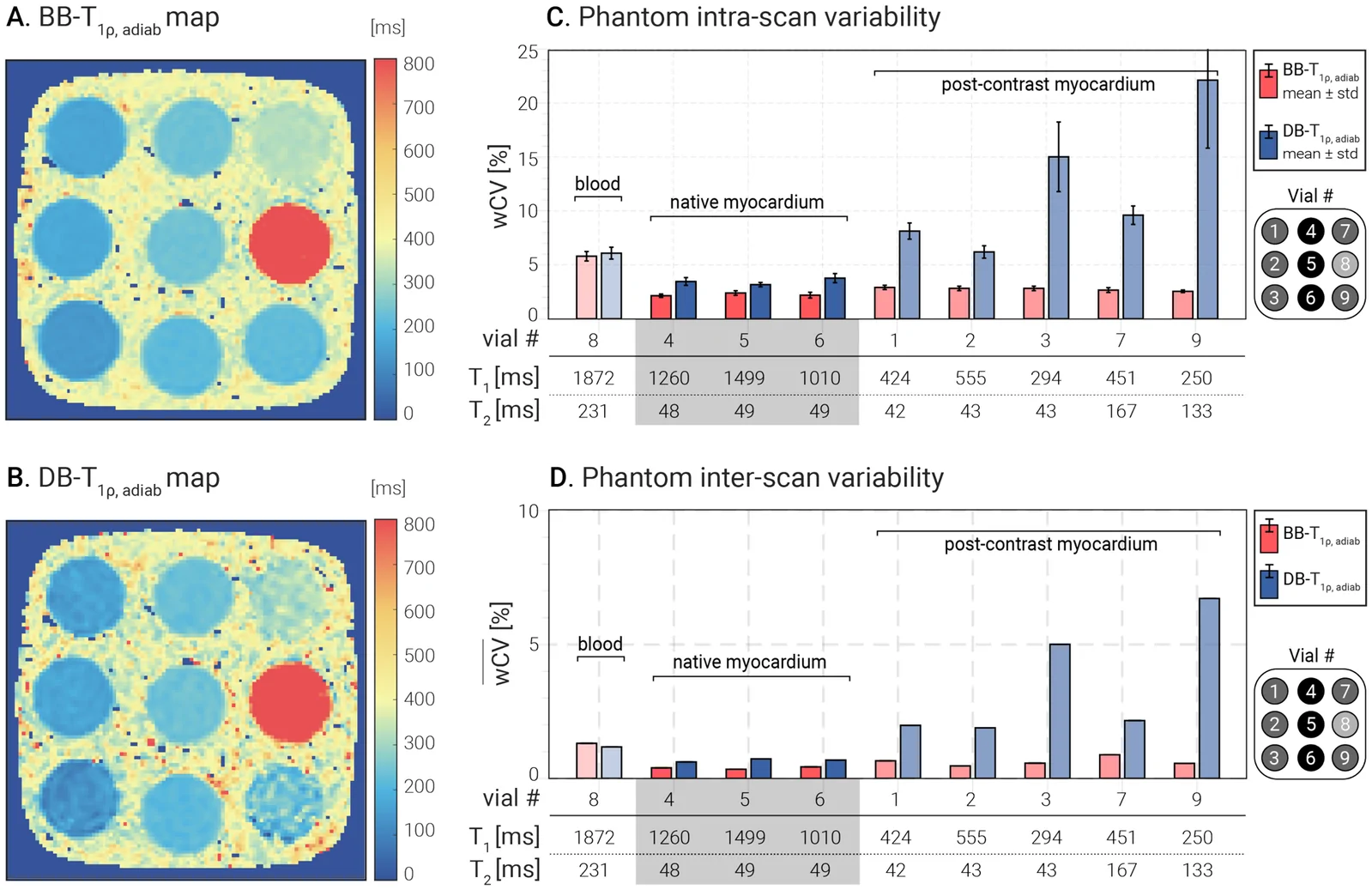
Representative (**A**) bright-blood (BB)- and (**B**) dark-blood (DB)-$T_{1\rho ,adiab}$ maps of the tissue-mimicking T1MES phantom. Good map quality was achieved with both BB and DB preparations, but higher variability can be observed in DB-$T_{1æ,adiab}$ maps, especially in vials #1–3, #7 and #9. (**C**) Intra- and (**D**) inter-scan variability measured for BB- and DB-$T_{1æ,adiab}$ mapping over 10 repetitions, reported for each vial. In native myocardium-mimicking vials (#4–6, highlighted in gray) a moderate increase in both intra- and inter-scan variability was observed for DB compared with BB-$T_{1æ,adiab}$ maps.

In the native myocardium-like vials (#4: $T_{1}$ = 1260 ms, #5: $T_{1}$ = 1499 ms, #6: $T_{1}$ = 1010 ms in Figure 3), DB-$T_{1\rho ,\;adiab}$ maps showed a moderate increase in intra-scan variability compared with BB-$T_{1\rho ,\;adiab}$ maps, as shown in Figure 3B (DB: $wCV_{i,r}$ = 3.46 ± 0.41%, BB: $wCV_{i,r}$ = 2.24 ± 0.23%, *p* < 0.01, $p_{adj}$ < 0.02). For shorter $T_{1}$, larger differences in intra-scan variability were observed with the DB-$T_{1\rho ,\;adiab}$ mapping technique (DB-$T_{1\rho ,\;adiab}$ $wCV_{i,r}$ = 12.21 ± 6.58%, BB-$T_{1\rho ,\;adiab}$ $wCV_{i,r}$ = 2.75 ± 0.23%, *p* < $10^{-4}$, $p_{adj}$ < $10^{-3}$) in vials #1 ($T_{1}$ = 424 ms), #2 ($T_{1}$ = 555 ms), #3 ($T_{1}$ = 294 ms), #7 ($T_{1}$ = 451 ms) and #9 ($T_{1}$ = 250 ms). This indicated a smaller dynamic range in the DB technique, following increased $T_{1}$ relaxation during the preparation delay. On the other hand, the blood-like vial (#8, $T_{1}$ = 1872 ms), characterized by very high $T_{1}$, yielded no significant difference in intra-scan variability between BB- and DB-$T_{1\rho ,\;adiab}$ maps (DB-$T_{1\rho ,\;adiab}$ $wCV_{i,r}$ = 6.08 ± 0.44%, BB-$T_{1\rho ,\;adiab}$ $wCV_{i,r}$ = 5.80 ± 0.55%, *p* = $p_{adj}$ = 0.06).

The inter-scan variability followed similar but attenuated trends as the intra-scan variability for both BB- and DB-$T_{1\rho ,\;adiab}$ values (myocardium-like vials #4–6: DB-$T_{1\rho ,\;adiab}$ $\bar{wCV_{i}}$ = 0.68 ± 0.06%, BB-$T_{1\rho ,\;adiab}$ $\bar{wCV_{i}}$ = 0.39 ± 0.04%, *p* = 0.07, $p_{adj}$ = 0.08; post-contrast tissue vials #1–3, #7 and #9: DB-$T_{1\rho ,\;adiab}$ $\bar{wCV_{i}}$ = 3.55 ± 2.20%, BB-$T_{1\rho ,\;adiab}$ $\bar{wCV_{i}}$ = 0.63 ± 0.16%, *p* = 0.04, $p_{adj}$ = 0.08). Neither difference remained significant after correction for multiple comparisons, indicating that, in contrast to the intra-scan variability, the increase in inter-scan variability with the DB technique did not reach statistical significance in the phantom. In the blood-like vial #8, a single value per technique was obtained (DB-$T_{1\rho ,\;adiab}$ $\bar{wCV_{i}}$ = 1.17%, BB-$T_{1\rho ,\;adiab}$ $\bar{wCV_{i}}$ = 1.31%) and no test was performed.

### 3.2. In Vivo $T_{1\rho ,adiab}$ Mapping Results

**Figure 4:**
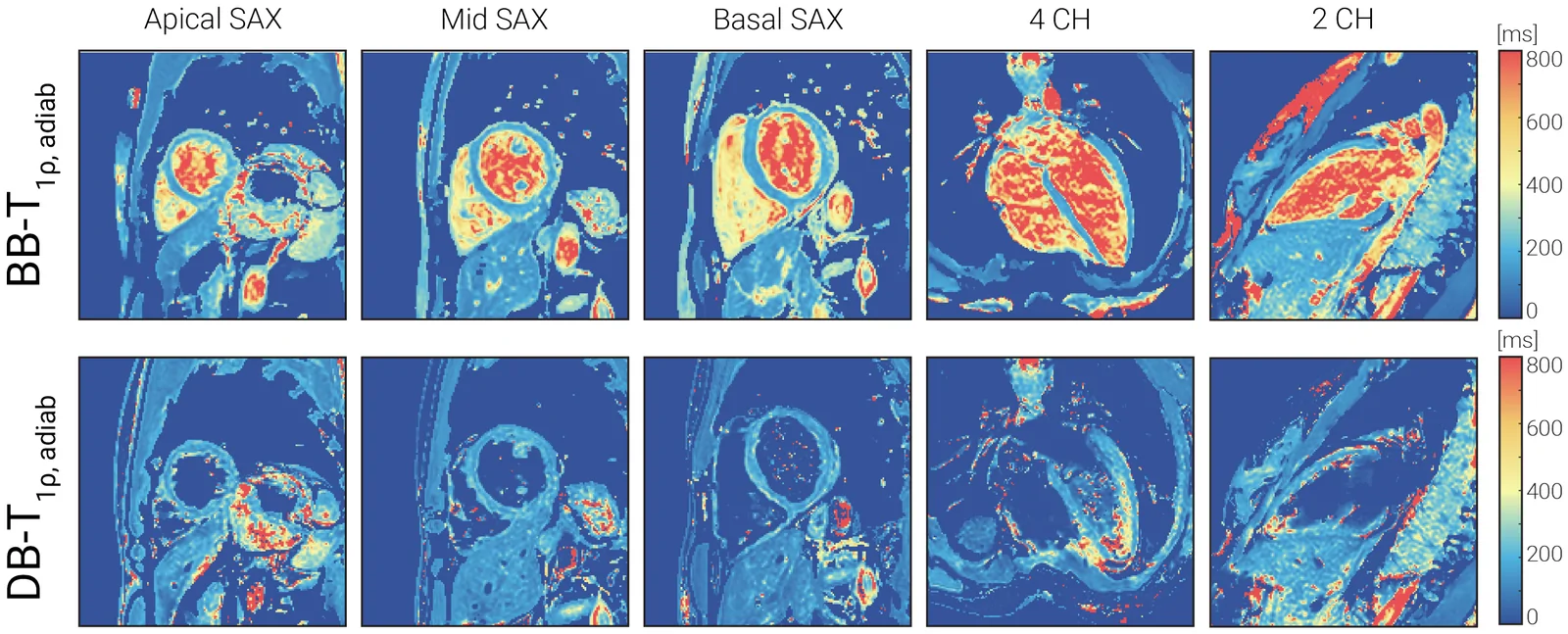
Mid short-axis (SAx), four chambers (4Ch) and two chambers (2Ch) bright-blood (BB)- and dark-blood (DB)-$T_{1\rho ,adiab}$ maps in a representative healthy subject. Both BB- and DB-$T_{1\rho ,adiab}$ maps achieved good visual map quality, with homogeneous values across the myocardium and no visible artifacts. DB-$T_{1\rho ,adiab}$ maps display thorough suppression of the signal from the blood pool in SAx and 2Ch view. Incomplete blood suppression is evident in the 4Ch view, where the primary blood flow is directed within the labeling slab.

Figure 4 displays BB- and DB-$T_{1\rho ,adiab}$ maps of a healthy volunteer in multiple orientations. Both DB and BB techniques show homogeneous $T_{1\rho ,adiab}$ values across the myocardium, with no visually apparent artifacts. The subject’s heart rate was on average 57 bpm, allowing a preparation delay δ = 610 ms and near-complete blood suppression in DB-$T_{1\rho ,adiab}$ maps. Only areas of stagnant flow present residual blood signal intensity, as evident in the apical region of the four-chamber and two-chamber views. Moreover, blood suppression appears less effective in the four-chamber view left ventricle, where the flow is primarily directed parallel to the slice encoding direction.

**Figure 5:**
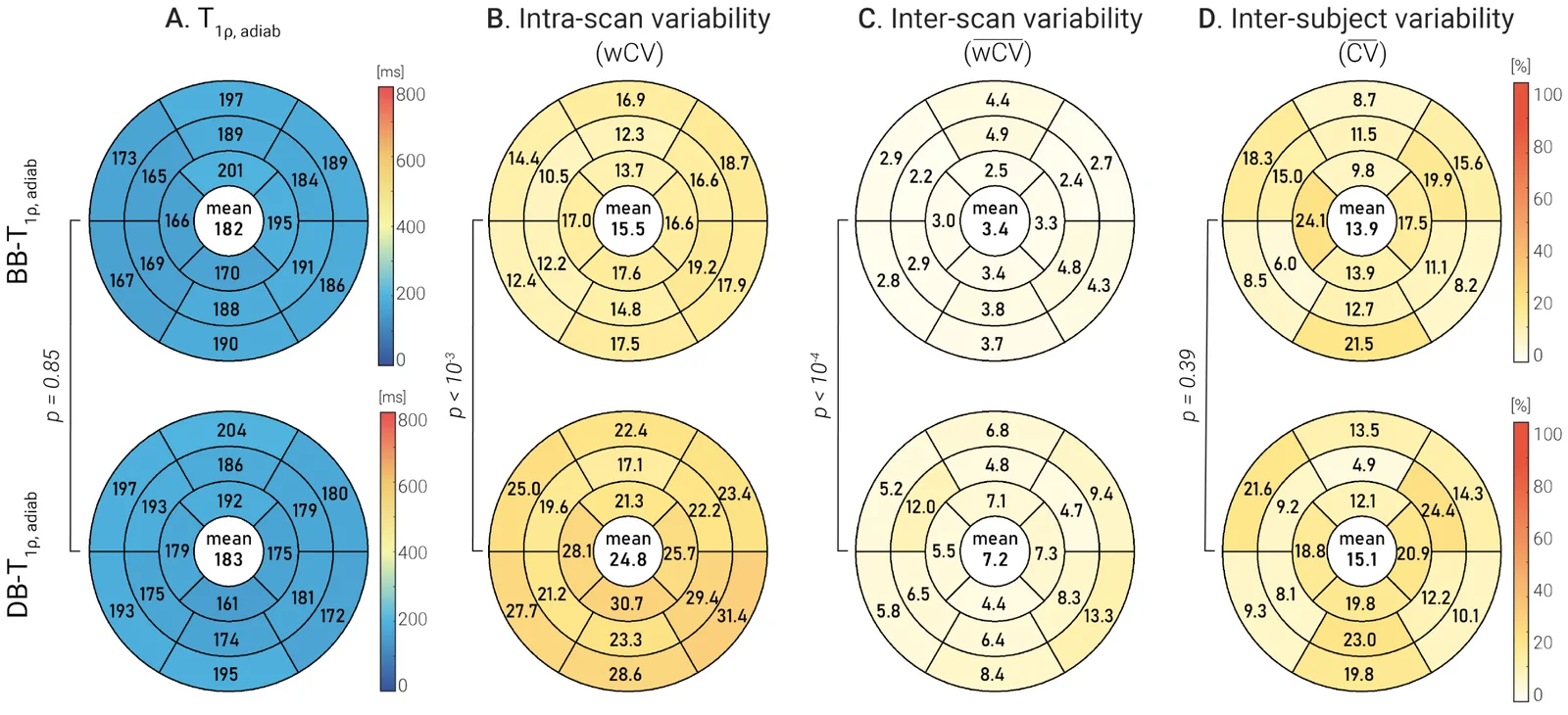
(**A**) Bullseye plots showing BB- and DB-$T_{1\rho ,adiab}$ values, averaged over all subjects and repetitions, for 16 AHA myocardial segments. Both BB- and DB-$T_{1\rho ,adiab}$ values are homogeneous across the 16 segments. No statistical difference was observed between BB- and DB-$T_{1\rho ,adiab}$ values. Bullseye plots reporting (**B**) the average intra-scan variability (wCV), (**C**) the average inter-scan variability ($\bar{wCV}$) and (**D**) the inter-subject variability ($\bar{CV}$) coefficients for BB- and DB-$T_{1\rho ,adiab}$ maps in 16 AHA myocardial segments.

No significant difference was found between BB- and DB-$T_{1\rho ,adiab}$ myocardial values across the healthy volunteers (p=0.85). Average BB-$T_{1\rho ,adiab}$ across all volunteers and all three short-axis slices was 182.32 ± 28.27 ms, compared with 183.49 ± 45.54 for DB-$T_{1\rho ,adiab}$. Bland–Altman analysis of the per-subject averages showed a bias of +0.58 ms, with 95% limits of agreement of −26.40 to +27.56 ms (Figure S2B). The near-zero bias indicates that blood suppression does not systematically alter the estimated relaxation times. Both BB- and DB-$T_{1\rho ,adiab}$ values were homogeneous across all myocardial segments, as shown in Figure 5. DB-$T_{1\rho ,adiab}$ maps showed visually improved myocardium-blood pool delineation. Additionally, blood signal suppression in the DB technique also allowed better visualization of the thin RV myocardial wall (Figure 4). The scanner-reported whole-body SAR values varied per subject, but were included in the 1.3–1.7 W/Kg range for both DB and BB-$T_{1\rho ,\;adiab}$ mapping sequences. These correspond to a range of 65% to 85% of the applicable whole-body SAR limit of 2.0 W/kg. SAR values were identical in DB and BB-$T_{1\rho ,\;adiab}$ sequences in all subjects.

DB-$T_{1\rho ,adiab}$ maps showed a moderate increase in intra-scan variability compared with BB-$T_{1\rho ,adiab}$ in vivo: $wCV_{i,r}$ = 15.51 ± 2.65 % for BB-$T_{1\rho ,adiab}$, $wCV_{i,r}$ = 24.82 ± 4.18 % for DB-$T_{1\rho ,adiab}$, $p<10^{-3}$. Similarly, inter-scan variability was higher for DB-$T_{1\rho ,adiab}$ maps ($\bar{wCV_{i}}$ = 3.38 ± 0.86 % for BB-$T_{1\rho ,adiab}$, $\bar{wCV_{i}}$ = 7.24 ± 2.55 % for DB-$T_{1\rho ,adiab}$, $p<10^{-4}$). Finally, a trend of increased inter-subject variability with DB-$T_{1\rho ,adiab}$ mapping was observed, but no statistical significance was observed ($\bar{CV}$ = 13.89 ± 5.27 % for BB-$T_{1\rho ,adiab}$, $\bar{CV}$ = 15.13 ± 6.05 % for DB-$T_{1\rho ,adiab}$, p=0.39).

#### 3.2.1. Blood Suppression Evaluation

**Figure 6:**
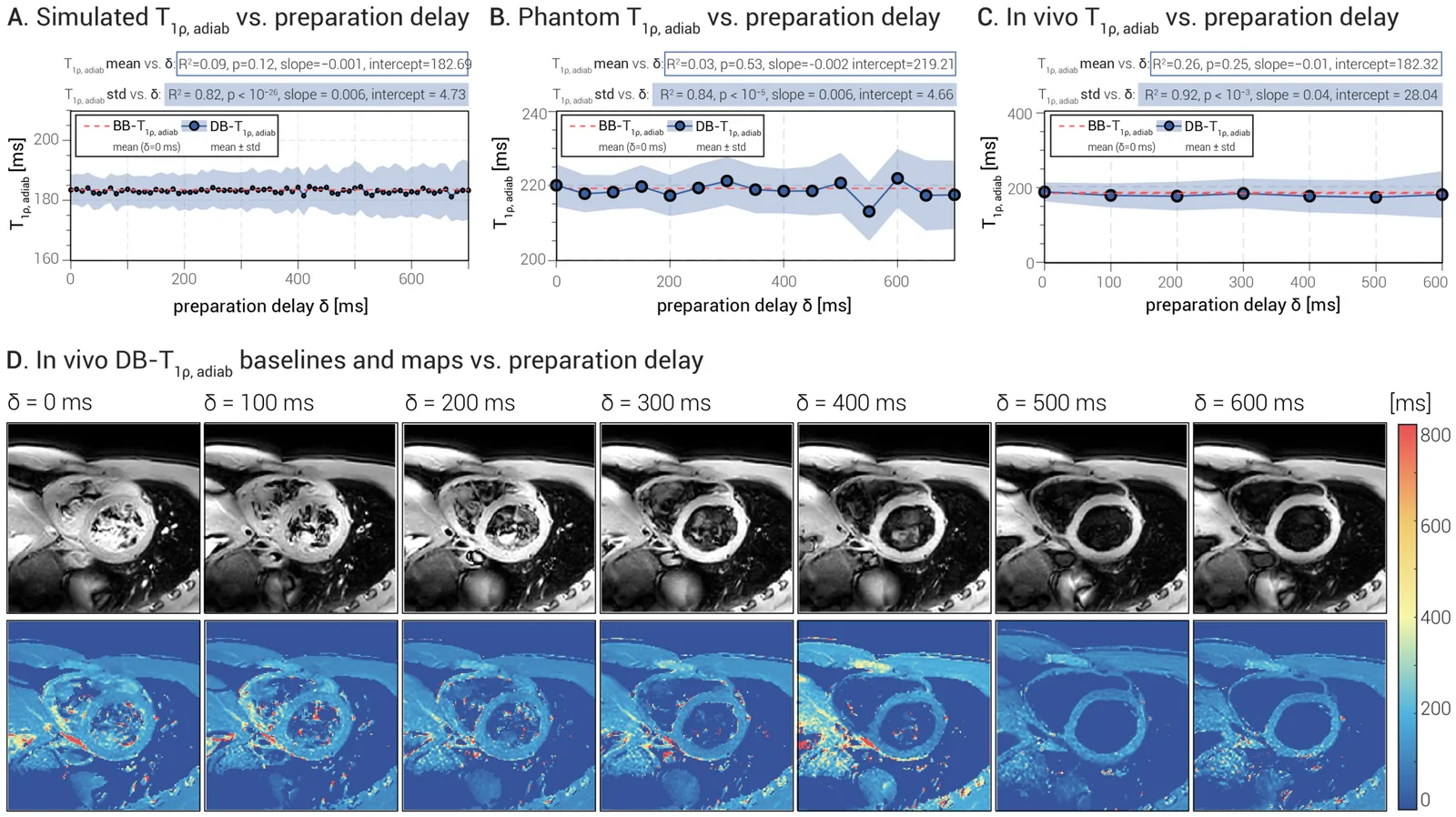
Measured dark-blood (DB)-$T_{1\rho ,adiab}$ values (blue dots) for varying preparation delay δ compared with reference bright-blood (BB)-$T_{1\rho ,adiab}$ technique (dashed red line) in (**A**) Bloch simulations ($T_{1}/T_{2}/T_{1æ,\mathrm{adiab}}$ = 1165/45/183 ms), (**B**) phantom imaging (vial #4: $T_{1}/T_{2}$ = 1260/48 ms) and (**C**) in vivo imaging in the mid-short-axis of a healthy volunteer (male, 27 y.o.). Mean DB-$T_{1\rho ,adiab}$ values remained approximately constant for increasing preparation delays. DB-$T_{1\rho ,adiab}$ standard deviation increased linearly with the preparation delay. (**D**) Example DB-$T_{1\rho ,adiab}$ baseline image (for $\tau_{SL}$ = 60 ms) and corresponding maps obtained in a single healthy volunteer for increasing preparation delays δ. At short delays clearly marked residual signal was obtained from the blood pool, while blood suppression visually improved for longer preparation delays.

In simulations, the estimated DB-$T_{1\rho ,adiab}$ value remained constant across various preparation delays δ, as the $T_{1}$ recovery experienced during the preparation delay is captured by the remaining two fit parameters *A* and *B* (see Supporting Materials Figure S1). Accordingly, Bloch simulation and phantom results show no trend in DB-$T_{1\rho ,adiab}$ for increasing preparation delays (simulations $R^{2}$ = 0.089, p=0.12, slope = −0.0011, intercept = 182.69; phantom $R^{2}$ = 0.03, p=0.53, slope = −0.0017, intercept = 219.21). Good agreement with the reference BB-$T_{1\rho ,adiab}$ sequence was achieved for all preparation delays, with deviations of less than 3 ms for all preparation delays, except for δ = 550 ms in phantom (Figure 6A,B). At the same time, the dynamic range is noticeably decreased for increasing preparation delays (Figure S1). Accordingly, increased standard deviation of the DB-$T_{1\rho ,adiab}$ values was observed for increasing preparation delay δ (simulations $R^{2}$ = 0.82, $p<10^{-26}$, slope = 0.0061, intercept = 4.73; phantom $R^{2}$ = 0.84, $p<10^{-5}$, slope = 0.0060, intercept = 4.66).

The same trends were observed in the myocardial DB-$T_{1\rho ,adiab}$ values obtained in a single healthy subject with different preparation delays (Figure 6C). The mean value showed no significant trend as a function of the delay time ($R^{2}$ = 0.26, p=0.25, slope = −0.011, intercept = 182.32) and good agreement with the BB-$T_{1\rho ,adiab}$ reference value (deviation < 16 ms for all preparation delays, Figure 6C). Intra-scan variability increased from 13.44% for 0 ms delay time to 26.25% at 600 ms delay time ($R^{2}$ = 0.92, $p<10^{-3}$, slope = 0.035, intercept = 28.04). The blood signal suppression was visually improved at longer delay times (Figure 6D). At short delays, substantial residual signal from the blood was obtained, particularly in areas of slow or stagnant flow, such as the right ventricular trabeculation or around the papillary muscles. At longer delay times, where more longitudinal magnetization recovery brings the signal of the inverted blood close to the zero crossing, complete blood suppression was observed. This was visible in both baseline images and maps (Figure 6D).

Blood-to-myocardium signal ratio was 3.18 ± 0.76 for the BB acquisition and 0.19 ± 0.11 for the DB acquisition in the mid-short-axis slice across the 10 healthy subjects. The reduction in blood-to-myocardium signal ratio achieved in DB-prepared images was statistically significant (Wilcoxon signed-rank test, $p<10^{-2}$).

#### 3.2.2. Myocardial Thickness Evaluation

**Figure 7:**
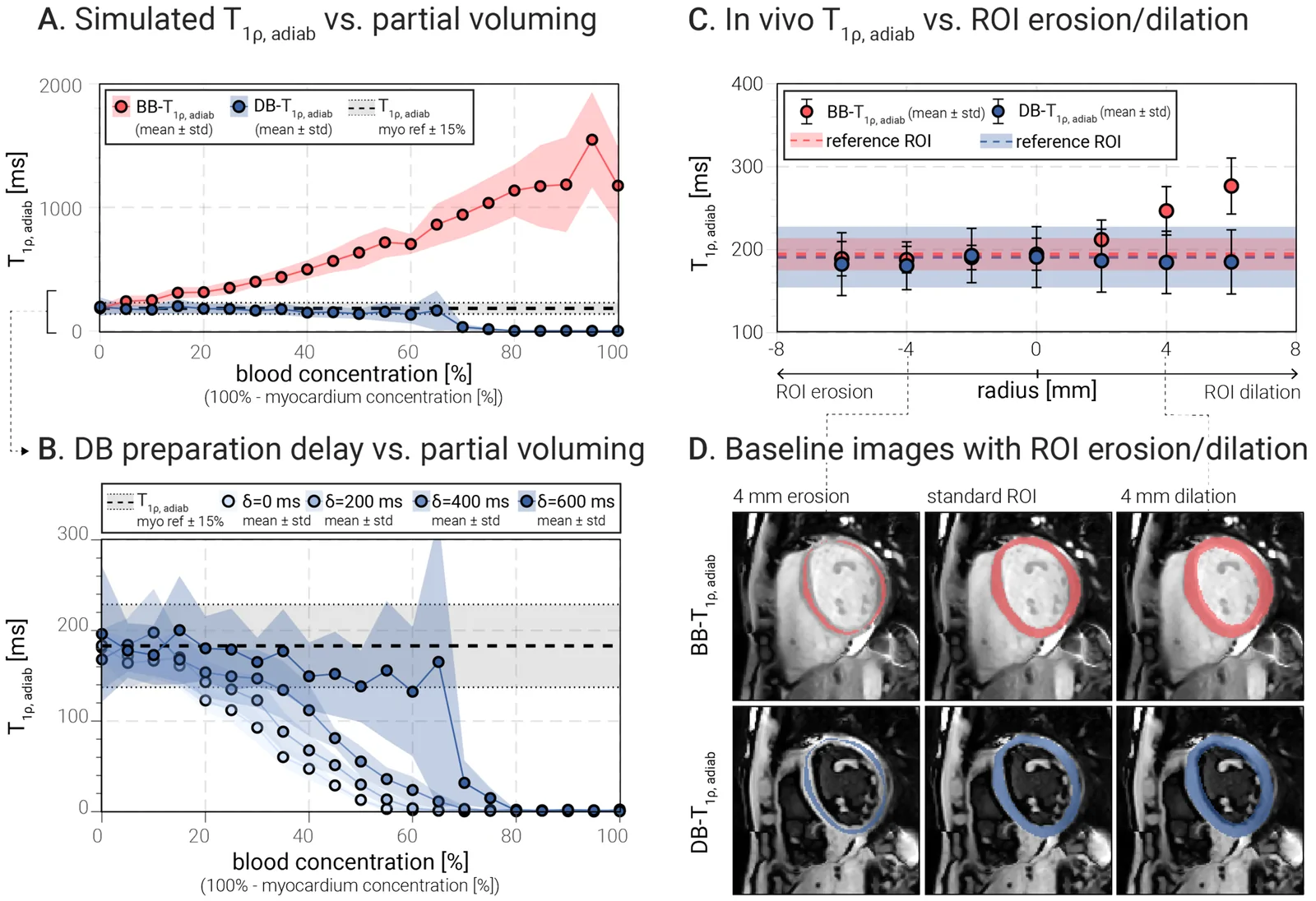
(**A**) Simulated bright-blood (BB)- (red) and dark-blood (DB)-$T_{1\rho ,adiab}$ (blue) values for varying voxel compositions (mean ± standard deviation) compared with the reference ± 15% deviation interval (gray shading) from the value obtained for 100% blood composition (black dashed line). BB-$T_{1\rho ,adiab}$ values showed a strong non-linear increase for increasing blood concentration in voxels with partial-voluming. DB-$T_{1\rho ,adiab}$ mapping showed reduced dependence on blood concentration. (**B**) Zoomed-in plot showing DB-$T_{1\rho ,adiab}$ values in the presence of partial-voluming for preparation delay δ = 0, 200, 400, 600 ms. Shorter preparation delays led to a progressively stronger dependence of DB-$T_{1\rho ,adiab}$ values on the blood concentration. (**C**) BB- and DB-$T_{1\rho ,adiab}$ values obtained in vivo by using (**D**) eroded and dilated myocardial ROIs (red for BB, blue for DB). DB-$T_{1\rho ,adiab}$ values remained within the reference interval for each case of dilation and erosion, while increasing BB-$T_{1\rho ,adiab}$ values were measured with ROI dilation.

Bloch simulations showed a strong non-linear increase of the BB-$T_{1\rho ,adiab}$ values with increasing blood concentration in voxels with partial-voluming (simulated BB-$T_{1\rho ,adiab}$ = 184 ms for 0% blood, 1386 ms for 100% blood, Figure 7A). Partial-voluming with more than 5% blood in a voxel, caused more than 15% deviation from the simulated $T_{1\rho ,adiab}$ time in the BB technique. On the other hand, DB-$T_{1\rho ,adiab}$ mapping showed reduced sensitivity to blood concentration. For a preparation delay of δ = 600 ms, deviations of less than 15% were observed in the presence of up to 65% blood concentration. Shorter preparation delays led to a progressively stronger dependence of DB-$T_{1\rho ,adiab}$ values on the blood concentration. With a blood concentration of 50% in the voxel, the DB-$T_{1\rho ,adiab}$ deviation from the 0% blood case was 88% for δ = 0 ms, 59% for δ = 200 ms, 31% for δ = 400 ms, and 6% for δ = 600 ms.

Figure 7C shows the BB- and DB-$T_{1\rho ,adiab}$ trends for eroded and dilated myocardial ROI in the healthy subjects cohort. Both BB- and DB-$T_{1\rho ,adiab}$ values vary less than 1 std from the value obtained for the automatic segmentation for all erosion cases. DB-$T_{1\rho ,adiab}$ values also remained within the reference interval defined by ±1 std for each case of dilation (DB-$T_{1\rho ,adiab}$ deviation from reference for 6 mm dilation: −7.15 ± 3.04%). Altered BB-$T_{1\rho ,adiab}$ values were observed for ROI dilation, with an increase of +16.48 ± 4.91% for a dilation radius of 2 mm and up to +47.8 ± 15.85% for a dilation radius of 6 mm.

**Figure 8:**
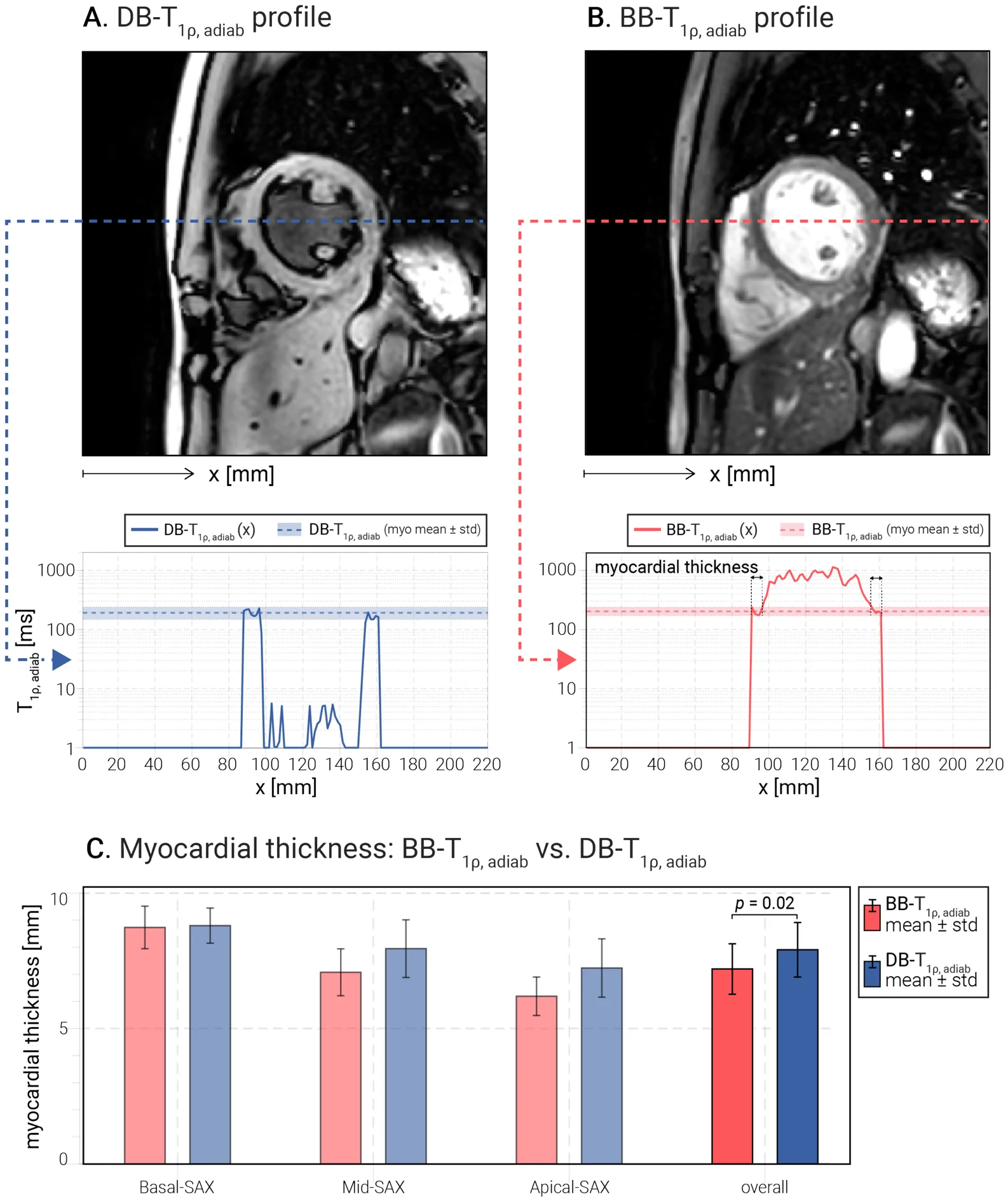
(**A**) Bright-blood (BB)- and (**B**) dark-blood (DB)-$T_{1\rho ,adiab}$ profile, illustrated along a horizontal line cutting through the center of the left ventricle, as shown on the corresponding baseline images. A sharp delineation at the myocardium interface is observed for DB-$T_{1\rho ,adiab}$, while the BB-$T_{1\rho ,adiab}$ profile presents a gradual transition between the myocardium and blood pool, with reduced apparent thickness of the apparent myocardium. (**C**) Average apparent myocardial thickness in BB- and DB-$T_{1\rho ,adiab}$ maps, measured as the width of the $T_{1\rho ,adiab}$ profile plateau for 5° circular sectors and averaged over the 72 sectors around the entire myocardium. Overall, DB-$T_{1\rho ,adiab}$ maps yielded a +9.86 ± 2.27% increase in myocardial thickness compared with BB-$T_{1\rho ,adiab}$ (p=0.02).

Figure 8A,B depicts the BB- and DB-$T_{1\rho ,adiab}$ profile, plotted along a row of pixels cutting horizontally through the center of the left ventricle in a representative healthy subject’s mid-short-axis slice. BB-$T_{1\rho ,adiab}$ maps show a gradual transition of the myocardium to the blood pool. Contrarily, a sharp delineation at the myocardium interface is observed for DB-$T_{1\rho ,adiab}$. The average apparent myocardial thickness in DB-$T_{1\rho ,\;adiab}$ maps across all subjects and slices, measured as the width of the plateau in the profile plot, shows a +9.86 ± 2.27% increase compared with BB-$T_{1\rho ,\;adiab}$ maps (p=0.02, Figure 8C). This quantity denotes the extent of myocardium over which $T_{1\rho ,\;adiab}$ can be quantified without relevant blood partial-volume contamination, and does not represent a measurement of the true anatomical wall thickness. Specifically, DB-$T_{1\rho ,adiab}$ apparent myocardial thickness was significantly higher than the corresponding BB-$T_{1\rho ,adiab}$ case for mid (BB: 7.08 ± 1.74 mm, DB: 7.95 ± 1.53 mm) and apical (BB: 6.19 ± 1.82 mm, DB: 7.23 ± 1.90 mm) short-axis slices. BB and DB apparent myocardial thickness values were not significantly different in the basal short-axis slice (BB: 8.73 ± 2.16 mm, DB: 8.80 ± 2.31 mm).

#### 3.2.3. Patients $T_{1\rho ,adiab}$ Mapping Results

**Figure 9:**
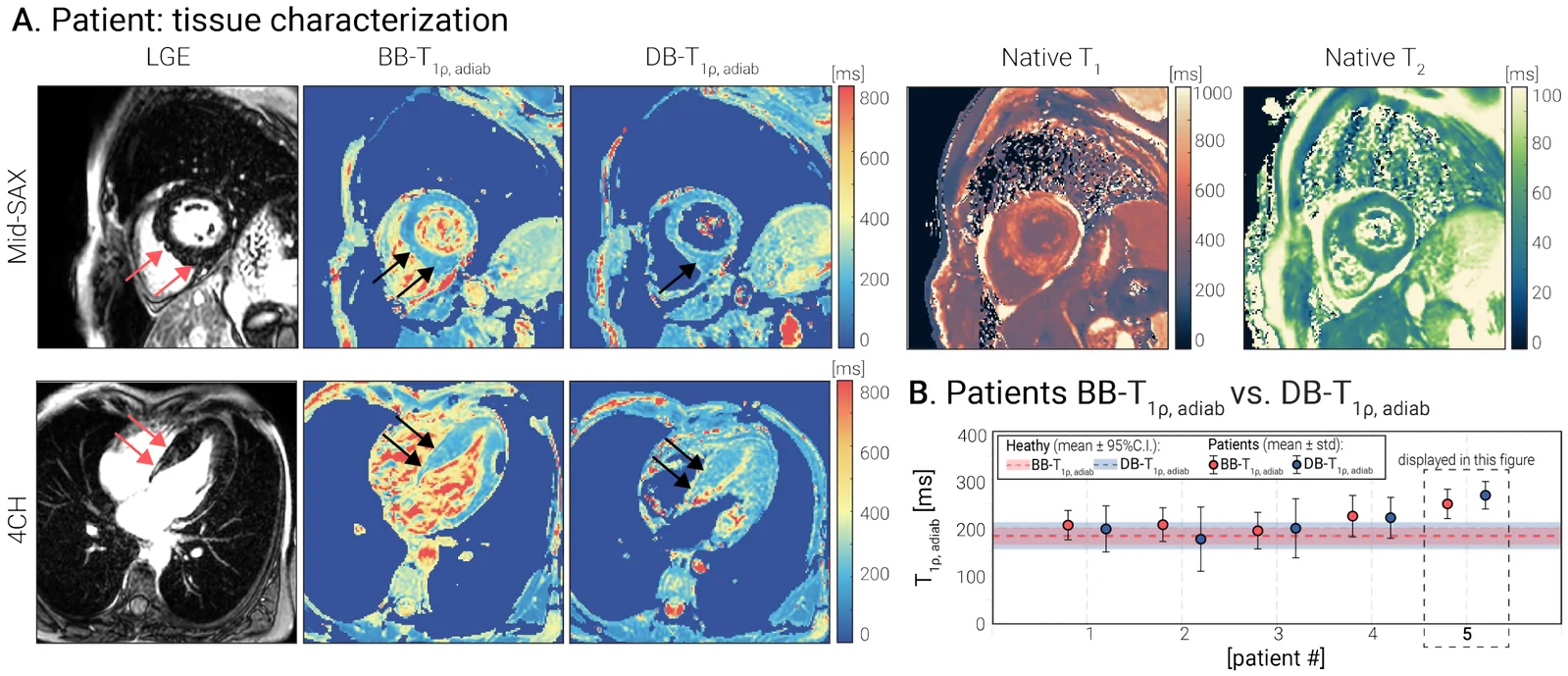
(**A**) Late gadolinium enhancement (LGE), bright-blood (BB)-, dark-blood (DB)-$T_{1\rho ,adiab}$, $T_{1}$ and $T_{2}$ images of a 47-years-old female patient (#5) suffering from hypertrophic cardiomyopathy. Black arrows indicate areas of suspected fibrosis, co-localized with areas of LGE enhancement (red arrows). LGE images show diffuse mid-wall myocardial enhancement in the mid-to-apical areas of myocardial septal hypertrophy (red arrows). BB-$T_{1\rho ,adiab}$ and DB-$T_{1\rho ,adiab}$ maps show $T_{1\rho ,adiab}$ elevation corresponding to LGE positive regions. Residual off-resonance artifacts are visible in the infero-lateral segment of the BB- and DB-$T_{1\rho ,adiab}$ short-axis maps and in the apical lateral BB-$T_{1\rho ,adiab}$ 4Ch segment. The native $T_{1}$ map also show diffuse and speckled enhancement, partially co-localized with LGE-positive areas, whereas the native $T_{2}$ map did not show visually discernable areas of enhancement. (**B**) Mean BB-$T_{1\rho ,adiab}$ (red) and DB-$T_{1\rho ,adiab}$ (blue) mid-SAx myocardial values ± their standard deviation for all five patients compared with the mean ± 95% confidence interval (dashed line) reference range obtained in healthy volunteers. Patient #5 (shown in (**A**)) displays increased BB-$T_{1\rho ,adiab}$ and DB-$T_{1\rho ,adiab}$ values above the reference 95% C.I. of healthy subjects, in agreement with the diagnosed diffuse LGE mid-wall enhancement caused by hypertrophic cardiomyopathy.

The patient cohort was acquired to assess the technical feasibility of BB-$T_{1\rho ,\;adiab}$ and DB-$T_{1\rho ,\;adiab}$ mapping in a clinical setting; given the cohort size and the single LGE-positive case, the following observations are illustrative and do not permit any conclusion on diagnostic performance. Two of the seven scanned patients (29%) were excluded because of severely compromised image quality in all acquired scans, including the clinical $T_{1}$, $T_{2}$ and LGE images, caused by excessive motion or poor RF shimming. Specifically, one patient failed to comply with breath-holding instructions, while significant $B_{0}$ artifacts corrupted all quantitative maps acquired in the second patient, likely due to poor shimming. The degradation was thus not specific to the $T_{1\rho ,\;adiab}$ acquisitions. Of the remaining five patients, one subject was LGE-positive, with signs of fibrosis in the LV myocardium (see Supporting Materials Table S1). Figure 9A shows the BB- and DB-$T_{1\rho ,adiab}$ maps acquired in the LGE-positive patient (female, 47 y.o.), who suffered from asymmetrical hypertrophic cardiomyopathy (HCM). The patient presented diffuse mid-wall myocardial enhancement in the areas of hypertrophy (Figure 9A). Native $T_{1}$ maps show diffuse and speckled enhancement, partially co-localized with LGE-positive areas. Native $T_{2}$ maps did not show visually discernable areas of enhancement in the myocardium. Overall decreased visual BB- and DB-$T_{1\rho ,adiab}$ map quality was observed compared with the healthy subjects due to different scanner hardware characteristics, such as the single transmit channel and the lack of advanced shim modes. Moreover, higher heart rates and heart rate variability, as well as reduced compliance to breath holding protocols could have negatively impacted image quality in patients with respect to healthy subjects. An off-resonance artifact is visible in the infero-lateral segment of the BB- and DB-$T_{1\rho ,adiab}$ short-axis maps and in the apical lateral DB-$T_{1\rho ,adiab}$ 4Ch segment, due to incomplete shimming. Yet, comparable image quality was observed between BB- and DB-$T_{1\rho ,adiab}$ maps. Elevated $T_{1\rho ,adiab}$ values were measured, spatially coinciding with the LGE positive areas. Global mean BB- and DB-$T_{1\rho ,adiab}$ values in this patient were elevated compared with the rest of the patient cohort and fell above the 95% confidence interval of the reference BB- and DB-$T_{1\rho ,adiab}$ values obtained in the healthy subjects (patient #5: BB-$T_{1\rho ,adiab}$ = 249.98 ± 61.34 ms, DB-$T_{1\rho ,adiab}$ = 268.01 ± 57.44 ms; Figure 9B). Residual blood pool signal is observed in the mid-LV blood pool, as the high heart rate of the patient, limited the maximum allowed preparation delay to δ = 480 ms.

## 4. Discussion

In this study, we proposed an adiabatic $T_{1\rho ,adiab}$ mapping technique with intrinsic dark-blood contrast for myocardial tissue characterization at 3T. Dark-blood contrast was achieved through a combination of selective and non-selective AFPs, achieving $T_{1\rho ,adiab}$ weighting within the labeling slab and signal inversion outside. In simulations, where the ground-truth relaxation time is known, the proposed DB-$T_{1\rho ,\;adiab}$ preparation yielded unbiased $T_{1\rho ,\;adiab}$ estimates across all preparation delays. In phantom and in vivo, the Bland–Altman analysis revealed that DB-$T_{1\rho ,\;adiab}$ values were in agreement with the BB-$T_{1\rho ,\;adiab}$ reference. Imaging in healthy volunteers and a small clinical, proof-of-principle cohort showed comparable mapping quality to BB contrast, with improved delineation of the myocardium/blood pool interface.

DB-$T_{1\rho ,adiab}$ values were in line with BB-$T_{1\rho ,adiab}$ values reported in this and previous studies [25], both in phantoms and in vivo. BB and DB preparations employ an identical RF pulse train and differ only in the slab-selection gradient applied during all but the final AFP pulse. The RF power deposition of the two preparations is consequently identical, and remained below the whole-body SAR limit of 2.0 W/Kg for all subjects. SAR scales with the number of AFP pulses in the preparation, which limits the maximum attainable spin-lock duration, and depends on subject size. The preparation delay was adjusted per subject depending on the heart rate, ranging from 395 to 745 ms. As the additional $T_{1}$ recovery during the delay is absorbed by the parameters *A* and *B* of the three parameter model, this subject-specific variation was not expected to bias the $T_{1\rho ,\;adiab}$ estimate, which is supported by the absence of a significant trend of $T_{1\rho ,\;adiab}$ with δ in simulation, phantom, and in vivo (Figure 6). However, the effective preparation delay affected the achievable precision and the degree of blood suppression.

Dark-blood contrast in myocardial mapping has previously been achieved with dedicated preparation modules placed before the quantitative preparation, most commonly DIR or MSDE. Both approaches suppress the blood signal effectively, but they do so at a cost that the proposed method avoids. DIR preparations require an additional inversion module and an inversion time matched to the blood $T_{1}$, which lengthens the sequence, restricts the available time within the cardiac cycle, and makes the achieved suppression sensitive to the assumed blood $T_{1}$. Additionally, the relaxation-based suppression also acts on intra-myocardial blood. MSDE methods also require an additional preparation block and introduce diffusion-weighting. In the proposed approach, dark-blood contrast is generated within the existing spin-lock preparation by applying a slab-selection gradient to all but the final AFP pulse. The only cost in sequence timing is the preparation delay, which is played within the same cardiac cycle. As the mechanism is based on cross-slice inflow rather than on relaxation, intra-myocardial blood is not expected to be affected, which is consistent with the agreement between BB and DB myocardial values observed in this work. We therefore expect the proposed method to be advantageous where scan time and SAR are constrained, and where the blood $T_{1}$ is not known reliably, for instance after contrast administration.

Simulation results displayed a differential behavior of DB-$T_{1\rho ,adiab}$ mapping in voxels with partial-voluming for different preparation delays. This trend appeared because the saturation baseline image was not acquired with dark-blood contrast. A combination of DIR and slice-selective saturation pulses could potentially be used to acquire the saturation-prepared image with dark-blood contrast. This warrants further investigation to eliminate residual dependence on the preparation delay and further increase the robustness of the DB-$T_{1\rho ,adiab}$ mapping technique in the presence of partial-voluming.

Increased preparation delays δ in the DB technique led on the one hand to compromised precision, because of the reduced dynamic range, and on the other hand to improved blood signal attenuation, due to the longer $T_{1}$ recovery after inversion. Hence, the choice of the preparation delay δ implicates a trade-off between variability and blood suppression. However, our results indicated that complete blood signal nulling is not necessary to achieve resilience against blood contributions in the voxel. Considering $T_{1}$ = 1932 ms [57] and $T_{1\rho ,adiab}$ = 934 ms for blood, a preparation delay δ≈ 1300 ms is required for complete blood nulling, which exceeds the typical RR duration. Nonetheless, good blood suppression was observed in vivo DB-$T_{1\rho ,adiab}$ maps already at intermediate preparation delays (δ> 500 ms). As the blood pool only experiences a single AFP pulse across the three DB-$T_{1\rho ,adiab}$-prepared dynamics its signal intensity remains largely constant. The constant blood signal is then captured in the *B* term of the three-parameter fit model, without biasing the estimation of the blood DB-$T_{1\rho ,adiab}$ (Supporting Materials Figure S1). Too low preparation delays, however, can compromise the precision of the DB technique in voxels with partial-voluming. Here, strong residual blood pool intensity can render the fitting susceptible to random fluctuations caused by beat-to-beat differences in flow patterns. Thus, careful consideration of the preparation delay is required to optimize the interplay between blood signal suppression and quantification precision. For practical use, we recommend selecting the preparation delay as the longest value compatible with end-diastolic acquisition within the available R-R interval, capped at approximately 700 ms, beyond which the loss of precision outweighs the additional blood suppression. At elevated heart rates the attainable delay is shortened, as illustrated by the patient acquired at δ = 480 ms, and residual blood signal in the ventricular cavity should be expected. The resulting maps remain quantitatively valid, since the estimated $T_{1\rho ,\;adiab}$ is independent of the delay, but the benefit for sub-endocardial delineation is reduced. In the presence of marked heart-rate variability or arrhythmia, the delay should be derived from the shortest anticipated R-R interval to preserve end-diastolic acquisition. In regions of slow or stagnant flow, such as the apex, the right ventricular trabeculations and the vicinity of the papillary muscles, and in views in which flow is predominantly in-plane, such as the four-chamber view, suppression is inherently less effective and longer delays provide only limited benefit. Short-axis acquisition is therefore preferable where sub-endocardial assessment is the objective. Nevertheless, both intra-scan and inter-scan variability values remain markedly lower than those obtained with conventional continuous-wave spin-lock preparations at 3T using the same metric definitions (precision 37.61 ± 19.42%, reproducibility 47.39 ± 12.06%) [25], indicating that the variability of the proposed technique lies within the range previously considered acceptable for myocardial $T_{1\rho }$ mapping at this field strength. A comparable trade-off has been reported for established dark-blood mapping approaches. In MSDE-based dark-blood native $T_{1}$ mapping at 3T, myocardial $T_{1}$ times were statistically indistinguishable from the bright-blood reference (p=0.20), while precision degraded by a factor of approximately two (63.1 ± 6.4 ms versus 133.9 ± 24.6 ms) [40], compared with a factor of approximately 1.6 in the present work.

DB-$T_{1\rho ,adiab}$ mapping yielded slightly increased apparent myocardial thickness compared to BB-$T_{1\rho ,adiab}$. Furthermore, due to the reduced effects of partial-voluming, increased resilience towards ROI size was observed. This trend is in agreement with previous DIR-based DB-$T_{2}$ mapping studies, reporting both visually and numerically larger (+1.43 mm) myocardial thickness in DB maps compared with BB maps [58,59]. Increased thickness may aid in the reliable quantification in the presence of thin myocardiums, such as in the RV of patients with dilated cardiomyopathy (DCM). It can also reduce the intra-reader variability caused by variations in the placement of the myocardial ROI. Beyond the visual and ROI-based assessment presented here, dark-blood $T_{1\rho ,\;adiab}$ maps are of particular interest as an input to automated myocardial tissue-characterization pipelines. Automated segmentation of the myocardium is a prerequisite for large-scale quantitative analysis, and partial-volume contamination at the endocardial border represents a source of error in both the delineation of the contour and the quantitative values extracted from it. By suppressing the blood signal within the $T_{1\rho ,\;adiab}$ preparation itself, the proposed technique yields a sharp myocardium-blood interface and values that are substantially less sensitive to the precise position of the endocardial contour, as demonstrated by the erosion and dilation analysis. Deep-learning-based segmentation and detection methods have been applied extensively to MR images across anatomies [53,54,60], and improving the intrinsic contrast at the tissue boundary is complementary to such algorithmic developments. Combining dark-blood quantitative mapping with automated analysis is therefore a promising direction for future work. Importantly, the improved visualization of the blood myocardium interface may enable reliable evaluation of the sub-endocardium. This can be critical for the visualization and assessment of characteristic sub-endocardial scars [46]. Thus, the proposed DB technique may help distinguish scar-related myocardial $T_{1\rho ,adiab}$ hyperenhancement from $T_{1\rho ,adiab}$ enhancement caused by blood signal contributions. Further evaluation of the proposed technique in subjects with subendocardial scars in comparison to DB LGE techniques is warranted to assess its clinical use in this patient population.

The Bloch simulations presented here assume stationary compartments and complete inversion of the blood magnetization outside the labeling slab. Consequently, they characterize the quantification behavior of the preparation but not the efficacy of the blood suppression itself. In vivo the blood suppression also depends on blood velocity, flow direction relative to the imaging slice, labeling-slab thickness, and the inflow time available within the cardiac cycle. These effects were assessed experimentally in this work. The observed reduction of blood suppression in regions of slow or stagnant flow, and in views with predominantly in-plane flow, is consistent with the flow-dependent nature of the mechanism. A flow-resolved simulation framework would allow a more systematic optimization of the labeling slab and delay and represents an interesting direction for future work.

Moreover, the statistical comparisons presented in this study used the myocardial segment as the unit of analysis, after averaging over repetitions and subjects. Adjacent segments are spatially correlated and all subjects contribute to every segment, so the segment-wise values are not mutually independent, and the reported tests are best interpreted as descriptive of the spatial consistency of the observed differences rather than as inference on the population of subjects. A repeated-measures or mixed-effects framework would be required to resolve the individual contributions of subject, slice, and segment, which was beyond the scope of the present cohort.

A further consideration concerns the segmentation. The same automatic model was applied to BB-$T_{1\rho ,\;adiab}$ and DB-$T_{1\rho ,\;adiab}$ images despite their markedly different blood-myocardium contrast, which may in principle introduce a method-dependent bias in the delineation of the endocardial boundary. Two of the three analyses reported here are largely insensitive to this. First, the erosion/dilation experiment (Figure 7C) quantifies the effect of systematic contour displacement of up to 6 mm, well beyond any plausible segmentation error. Second, the apparent-thickness analysis derives thickness from the $T_{1\rho ,\;adiab}$ profile within circular sectors rather than from the contour itself. The segment-wise mean values, however, remain subject to a residual dependence on the segmentation, and small systematic differences between BB-$T_{1\rho ,\;adiab}$ and DB-$T_{1\rho ,\;adiab}$ contours cannot be excluded. A blinded multi-observer comparison reporting Dice score, boundary distance and inter-observer agreement separately for the two preparations would be required to quantify this effect, and was considered beyond the scope of the present work.

The initial proof-of-principle cohort demonstrated map quality that is comparable to BB-$T_{1\rho ,adiab}$. However, the clinical evaluation in this work is limited to a proof-of-principle cohort of five evaluable patients containing a single LGE-positive case. Accordingly, the observation of elevated $T_{1\rho ,\;adiab}$ values co-localized with LGE-positive myocardium in this patient is anecdotal, and no statement on the sensitivity or specificity of BB-$T_{1\rho ,\;adiab}$ or DB-$T_{1\rho ,\;adiab}$ mapping for the detection of scar or fibrosis can be derived from these data. Adequately powered studies in cohorts with pronounced and, in particular, sub-endocardial scar are required. Furthermore, healthy subjects and patients were scanned on two different 3T platforms at two different institutions. Consequently, the comparison of patient $T_{1\rho ,\;adiab}$ values with the reference range established in healthy subjects (Figure 9B) is confounded by hardware differences, in particular the single-transmit architecture and the reduced shimming capability of the system used for the patient scans, in addition to the physiological differences between the cohorts. This reference range should therefore be regarded as indicative. Cross-platform validation, ideally using a common phantom or traveling volunteers, is required before platform-independent reference values can be established. Moreover, the intrinsic clinical sensitivity of adiabatic BB- and DB-$T_{1\rho ,adiab}$ relaxation towards scar and fibrosis needs to be studied in relevant patient cohorts. Specifically, it warrants characterization in cohorts of patients with pronounced myocardial scars, such as chronic myocardial infraction patients. This is a crucial step to evaluate the clinical value of BB and DB adiabatic $T_{1\rho ,adiab}$ mapping and determine its potential use for non-contrast assessment of scar.

The clinical evaluation in this work is limited to a proof-of-principle cohort of five evaluable patients containing a single LGE-positive case. Accordingly, the observation of elevated $T_{1\rho ,\;adiab}$ values co-localized with LGE-positive myocardium in this patient is anecdotal, and no statement on the sensitivity or specificity of BB-$T_{1\rho ,\;adiab}$ or DB-$T_{1\rho ,\;adiab}$ mapping for the detection of scar or fibrosis can be derived from these data. Adequately powered studies in cohorts with pronounced and, in particular, sub-endocardial scar are required.

## 5. Conclusions

In this work, a novel adiabatic DB-$T_{1\rho ,adiab}$ preparation was proposed as an alternative to conventional BB-$T_{1\rho ,adiab}$ mapping for improved visualization of the myocardium. A combination of selective and non-selective AFP pulses, effectively suppressing the blood signal and maintaining adiabatic $T_{1\rho ,adiab}$ contrast in the myocardium was used to generate dark-blood contrast. Phantom and in vivo mapping results showed that the DB-$T_{1\rho ,\;adiab}$ preparation yielded myocardial $T_{1\rho ,\;adiab}$ values in agreement with the BB-$T_{1\rho ,\;adiab}$ reference, at the cost of moderately decreased precision. Thus, DB-$T_{1\rho ,adiab}$ mapping potentially represents an alternative to conventional BB-$T_{1\rho ,adiab}$ mapping to avoid high blood $T_{1\rho ,adiab}$ values overshadowing possible scar-related $T_{1\rho ,adiab}$ enhancement at the blood-to-myocardium interface.

## Data Availability

The data presented in this study are available on request from the corresponding author due to privacy and ethical restrictions.

## Supplementary Materials

The following supporting information can be downloaded at https://www.mdpi.com/article/10.3390/bioengineering13091031/s1, Figure S1: Simulated myocardial dark-blood (DB)-$T_{1\rho ,adiab}$ signal sampled at the different baseline points obtained for varying preparation delay; Table S1: Characteristics of the patients population; Figure S2: Agreement between bright-blood (BB)- and dark-blood (DB)-$T_{1\rho ,\;adiab}$ estimates; Figure S3: Voxel-wise standard deviation of the three-parameter fit for BB-$T_{1\rho ,\;adiab}$ (top row) and DB $T_{1\rho ,\;adiab}$ (bottom row) in apical, mid and basal short axis slices; Table S2: Heart rate and corresponding preparation delay (δ) for each subject.

## Abbreviations

The following abbreviations are used in this manuscript:

LGE	Late Gadolinium Enhancement
GBCA	Gadolinium Based Contrast Agent
RF	Radio-Frequency
SL	Spin-Lock
SAR	Specific Absorption Rate
AFP	Adiabatic Full Passage
DIR	Double Inversion-Recovery
MDSE	Motion-Sensitized Driven-Equilibrium
HS	Hyperbolic Secant
BB	Bright-Blood
DB	Dark-Blood
bSSFP	balanced Steady-State Free-Precession
WET	Water Suppression Enhanced through T_1_ effects
GraSE	Gradient Spin-Echo
LV	Left Ventricle
SAx	Short-Axis
2Ch	Two Chamber
4Ch	Four Chamber
DCM	Dilated Cardiomyopathy
ROI	Region Of Interest
