# Supplementary material for: Dark-Blood Adiabatic T1ρ Mapping of the Heart Using Combined Non-Selective and Slice-Selective RF Pulses at 3T
Source: Bioengineering (Basel). 2026 Sep 4;13(9):1031. doi: 10.3390/bioengineering13091031 (PMC13603637; doi:10.3390/bioengineering13091031)
Supplement: Supplementary file 1 [file bioengineering-13-01031-s001.zip › bioengineering-4440160-supplementary.pdf]

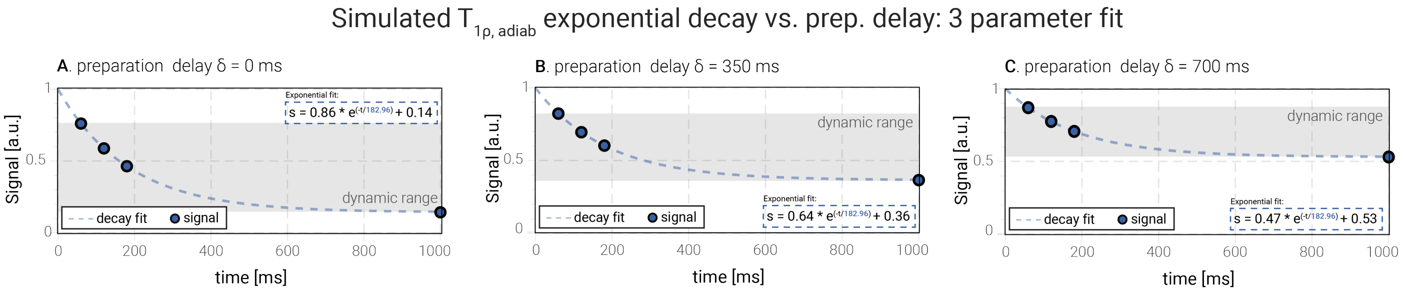

Figure S1. Simulated myocardial dark-blood (DB)-$T_{1æ,\mathrm{adiab}}$ signal sampled at the different baseline points (τ_{SL} = 60, 120, 180 ms and signal saturation) obtained for varying preparation delay (**A**) δ = 0, (**B**) δ = 350 and (**C**) δ = 700 ms. The corresponding exponential fit results are displayed by the dashed blue line. For increasing preparation delay, the DB-$T_{1æ,\mathrm{adiab}}$ estimation remained unbiased, but the measurement dynamic range (gray area) was reduced.

Table S1. Characteristics of the patients population.

**Patients characteristics**	
Gender [F/M]	5/0
Age [years]	46.80±10.32
BMI [kg/m^2]	28.07±7.33
HR [beats/min]	67.80±11.97
**CMR findings**	
LGE positive	1
**CMR diagnosis**	
Hypertrophic cardiomyopathy	1
Non-dilated hypokinetic cardiomyopathy	1
Negative CMR	3

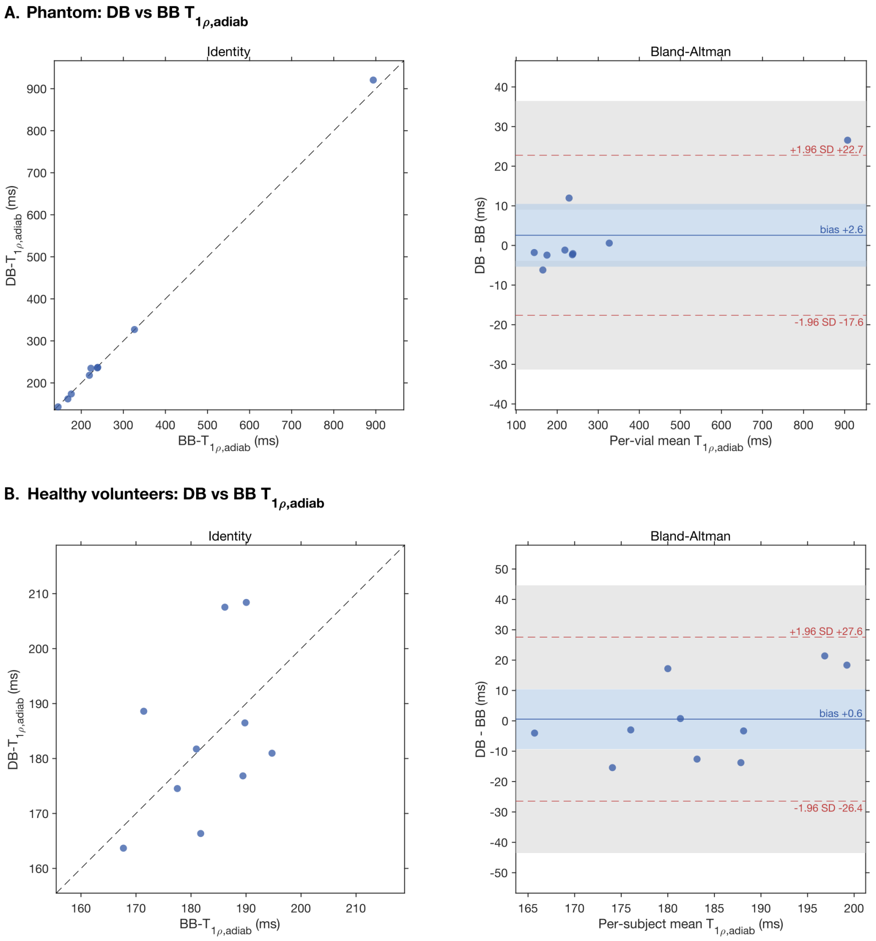

Figure S2. Agreement between bright-blood (BB)- and dark-blood (DB)-$T_{1\rho,adiab}$ estimates. (A) T1MES phantom, with each point representing one vial, averaged over 10 repetitions. (B) Healthy subjects, with each point representing one subject, averaged over all myocardial segments, slices and 3 repetitions. Left column shows identity plots, with the dashed line indicating the line of unity. Right column shows the corresponding Bland-Altman plots, showing the difference between the two techniques against their mean. The solid line denotes the bias (mean difference, DB-BB) and the dashed lines the 95% limits of agreement (bias $\pm$ 1.96 SD), with shaded bands indicating the corresponding 95% confidence intervals. In the phantom, DB-$T_{1\rho,adiab}$ estimation showed a bias of $+2.57$ ms with limits of agreement of -17.61 to $+22.74$ ms, corresponding to -5.2 to $+5.1$% of the measured values. In vivo, the bias was 0.58 ms with limits of agreement of -26.40 to +27.56 ms. The near-zero bias in both experiments indicates that the DB preparation does not systematically alter the estimated relaxation times, while the width of the limits of agreement reflects the reduced precision of the DB technique.

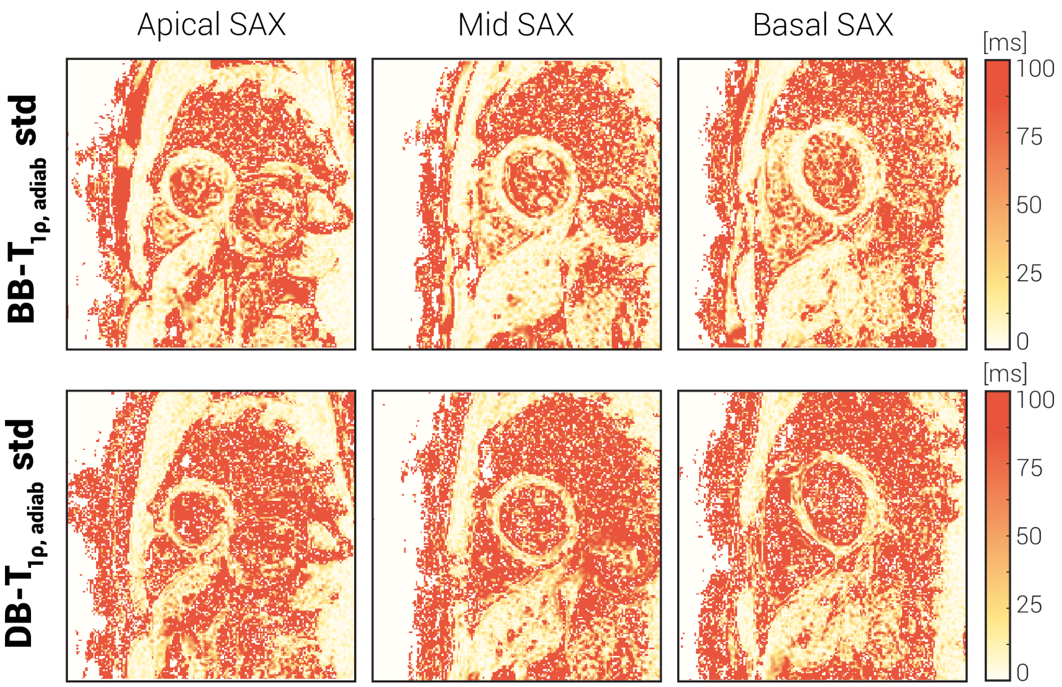

Figure S3. Voxel-wise standard deviation of the three-parameter fit for BB-$T_{1\rho,\mathrm{adiab}}$ (top row) and DB $T_{1\rho,\mathrm{adiab}}$ (bottom row) in apical, mid and basal short axis slices of the same healthy volunteer shown in Figure 4. Maps were obtained as the standard deviation of the fitted $T_{1\rho,adiab}$ returned by the mono-exponential three-parameter fit, and are displayed over a common range of 0-100 ms; voxels exceeding this range are shown saturated. Low fit uncertainty is confined to the myocardium in both preparations, whereas the elevated values in the left-ventricular blood pool of the DB acquisition reflect the intended suppression of the blood signal, which leaves no measurable decay to fit in those voxels. Non-finite fit outputs were set to zero prior to display.

Table S2. Heart rate and corresponding preparation delay (δ) for each subject.

**Healthy volunteers**			**Patients**		
	**HR**	**δ [ms]**		**HR**	**δ [ms]**
V01	60	560	P01	62	520
V02	60	560	P02	73	380
V03	56	630	P03	84	270
V04	60	560	P04	52	710
V05	71	400	P05	68	440
V06	57	610			
V07	50	745			
V08	57	610			
V09	54	665			
V10	72	395			
Mean±SD	59.70±6.95	573.50±108.60	Mean±SD	67.80±11.97,	464.00±165.02

The preparation delay was set to 600 ms by default in the protocol, and then adjusted in 10 ms shifts, depending on the subject hearth rate, to the longest delay possible before incurring in a conflict due to the limited cardiac cycle length.
